# Antibacterial and antibiofilm activity of platelet-rich plasma under different activation conditions against multidrug-resistant MRSA isolated from human skin abscesses

**DOI:** 10.1186/s12896-025-01078-x

**Published:** 2025-12-08

**Authors:** Asmaa Sayed Abdelgeliel, Waiel F. Sayed, Wesam M. Salem, Fatma S. Hassan

**Affiliations:** Department of Botany and Microbiology, Faculty of Science, South Valley University, Qena, 83523 Egypt

**Keywords:** MRSA, SSTIs, Antibiotic resistance, PRP, Antibacterial, Antibiofilm

## Abstract

**Background:**

This study evaluated the antibacterial efficacy of platelet-rich plasma (PRP), platelet-poor plasma (PPP), and three activated PRP fractions (APRP, PRP-T, and APRP-T) against multidrug-resistant (MDR) bacteria isolated from human cutaneous abscesses. The rise of multidrug-resistant microorganisms has created a need for the development of new infection treatment strategies. PRP was obtained from peripheral blood samples of healthy donors using an automated blood collection device known as platelet apheresis union. A total of 107 bacterial isolates were obtained from 102 pus samples collected from Egypt’s Qena General Hospital. The isolated bacteria were characterized using biochemical and serological studies, antibiotic susceptibility testing, and PCR detection of antibiotic resistance genes and virulence factors. The study aimed to evaluate the antibacterial, antibiofilm, and time-killing curve effects of PPP, PRP, APRP, PRP-T, and APRP-T against the MDR isolates.

**Results:**

The most prevalent strain was *Staphylococcus aureus* (79.35%), including methicillin-resistant (MRSA, 35.87%). Multidrug-resistance (MDR ≥ 3) was observed in various bacterial isolates, particularly against β-lactam antibiotics. Among the MRSA isolates, 100% tested positive for the virulence genes *icaD*,* LukED*, and *clfA*, as well as the antibiotic-resistant gene *mecA*. *S. aureus* strains, 31.51% were found to produce enterotoxins (ACD). Furthermore, 79.35% of *Staphylococcal* isolates had the ability to form biofilms. The active PRP fractions (APRP and APRP-T) exhibited antibacterial activities against MRSA strains, in both solid and liquid media as clear zone range (11.0–18.0 mm) and complete inhibition by (2730 ± 70 and 2180 ± 30 × 10^9^ cells/L). Significant antibiofilm activity was observed in the PPP, PRP, and PRP active fractions (APRP PRP-T, and APRP-T), resulting in a sharp decrease in MRSA biofilm optical density (OD) after treatment compared to control. The killing dynamics of APRP and APRP-T showed a steady decline in bacterial CFU after 4 h of treatment, followed by a dramatic decline after 24 h.

**Conclusions:**

The research suggests that PRP active fractions, specifically APRP and APRP-T, are effective in reducing complications caused by bacterial skin infections, particularly abscesses. These fractions exhibit simultaneous antibacterial, antibiofilm, and regenerative activities. Activated PRP fractions, particularly APRP-T, could serve as promising adjunct therapies against MDR MRSA infections.

**Supplementary Information:**

The online version contains supplementary material available at 10.1186/s12896-025-01078-x.

## Background

Skin and soft tissue infections (SSTIs) are a global health concern, imposing significant burdens on individuals and healthcare systems [1]. Abscesses can have developed in various tissues and organs across the body, especially subcutaneous, soft, and adipose tissues [2]. *Streptococcus pyogenes* and methicillin resistant *Staphylococcus aureus* (MRSA) are the most common microbial culprits behind abscess formation [3]. Coryneform, the genus *Micrococcus*, and various Gram-negative bacteria are commonly believed to inhabit the skin [4]. MRSA is the primary cause of skin infections and is presently the most pressing global health issue [5]. *S. aureus* possesses numerous virulence factors, such as cell wall-associated exoproteins, toxins, and superantigens [6]. One of the significant factors contributing to the persistence of *S. aureus* strains in both the host organism and the environment is their ability to form biofilms. The production of polysaccharide intracellular adhesion (PIA), which is encoded by the ica operon, is typically associated with *S. aureus* biofilm formation [7]. *S. aureus* generates a dense biofilm composed of aggregates, forming a three-dimensional structure [8]. Slime and biofilm formation in *S. aureus* are linked to icaA and icaD, which are essential for intercellular adhesion and the development of a bacterial multilayer in biofilm production [9]. Antibiotic resistance in *S. aureus* is a major global concern, necessitating the exploration of alternatives [10]. The effectiveness of antibiotics against *S. aureus* diminishes over time. Eventually, they cease to be viable therapeutic options for treating diseases caused by *S. aureus* strains [11].

Platelet-rich plasma (PRP), an autologous concentrate of peripheral blood platelets, is widely used in regenerative medicine due to its growth factor content and biosafety [12]. Tissue repair techniques are in high demand globally to address a range of medical conditions, including osteoarthritis, complex and persistent wounds, as well as musculoskeletal and spinal diseases. The emergence of PRP-based autologous cellular therapies offers potential as supplementary treatments in regenerative medicine [13]. Advances in understanding PRP technology and bio-formulation principles have led to improved recommendations for research directions and indications [13]. Over the past two decades, PRP has garnered significant attention due to its frequent use in clinical settings and its potential for promoting regenerative healing and antibacterial effects [14]. Studies have shown that PRP can rapidly, safely, and significantly reduce postoperative infection levels and pain intensity in sternotomy wounds [12]. PRP therapy is based on the principle that platelet growth factor (PGF) supports three stages of wound repair and healing, including inflammation, remodelling, and proliferation [13]. PRP contains abundant of growth factors and cytokines that play a role in the body’s inflammatory response [13]. PRP can be introduced into a wound via injection or applied topically in gel form. Its antibacterial properties on wound pathogens were reported as bactericidal action [15]. platelets have the ability to bind, aggregate, and internalize the microorganism, and produce numerous of peptides that have a potent antimicrobial activity [16] They secret some antimicrobial peptides, alpha particles, peroxides, chemokines, and other substances, that have antibacterial effect kill or inhibit pathogens, or release antimicrobial effects via chemotaxis and activation of immune cells [17]. Platelets has multiple roles in host defense by generating oxygen metabolites as hydrogen peroxide, superoxide and hydroxyl free radicals [18].

This study aims to investigate the antibacterial and antibiofilm potentials of PRP, which is considered a novel, cost-effective, and safe treatment option for human SSTIs. Additionally, we aim to discover new PRP activation methods to enhance its antibacterial effect, with the ultimate goal of developing it into a drug for the treatment of skin abscesses.

## Methods

### Sample collection and characterization

Pus samples (*n* = 102) were aseptically collected via sterile cotton swabs or syringe aspiration from skin infection including abscesses, purulent wound discharge, and infected hematomas or burns at Qena General Hospital, Egypt (February 2020–October 2021). Samples were collected in accordance with standard protocols and ethical guidelines approval [19]. We collected pus samples from skin (abscesses, wound discharge, and infected hematomas or burns). key patient demographics about social characteristics, such as sex and age were gathered. We labelled each specimen clearly and transported on dry ice to the microbiology laboratory until processed for Gram staining and culturing.

Informed consent to participate was obtained from all participants in this study. The study was conducted in accordance with the principles of the Declaration of Helsinki [19], and the protocol was approved by the Research Ethics Committee, Faculty of Science, South Valley University, Qena, Egypt, (REC-FScSVU) with ethics reference number (002/07/24).

### Isolation and identification of skin infection bacteria

Samples were aseptically streaked directly on blood agar (Oxoid^®^) and then incubated at 37 °C for 24 to 48 h. Different colonies were picked up and restreaked on agar media (mannitol salt agar, tryptic soy agar, blood agar) for purification then picked up in tryptic soy broth. After 24 h of pure isolate growth, bacterial isolates were kept with 70% glycerol/media at -80 °C for further identification. The characterizations of isolates were performed based on Gram staining, microscopic examination, colony characteristics, and biochemical assays were used to identify all suspected isolates as described in Bergey’s Manual of Determinative Bacteriology [20] and the rules of MacFaddin [21].

### Coagulase and DNase activities of *Staphylococcus aureus*

Pure colonies of isolates were added to Wassermann tubes containing 0.5 mL of sterile rabbit plasma or human plasma and then incubated at 37 °C for 4 h. Subsequently, the tubes were inspected for the formation of clots (fibrin clot formation), if the clot does not occur the tubes were incubated at room temperature for additional 18 h, Positive (plasma plus *S. aureus* strain) and negative (plasma only) controls were included [22]. For DNase activities, the DNase agar plates were inoculated with suspected colonies by spotting them onto small sections of the plates. We incubated the plates at 37 °C for 18 h. Subsequently, plates were flooded with regular hydrochloric acid (1 N), leading to the precipitation of DNA and causing the plates to become cloudy. The presence of a clear zone around the colony indicated the production of DNase [23].

### Serotyping of *Staphylococcus aureus* enterotoxin

The Reserve Passive Latex Agglutination technique (RPLA) using Kits for detects *Staphylococcal* enterotoxins A, B, C, and D (SET-RPLA, Denka Sekeu LTD, Japan) according to manufacture instructions [24].

### Detection of methicillin resistance *Staphylococcus aureus* (MRSA) isolates

Methicillin susceptibility of *S. aureus* was assessed using the disk diffusion method as described by Bonjean et al. [25]. Initially, the bacterial suspension adjusted to 0.5 MacFarland standards using 0.9% NaCl, then the suspension was spread on Mueller-Hinton agar surface, followed by the placement of a cefoxitin (30 µg) antibiotic disc on the inoculated plate. The plate was then incubated at 37 °C for 20–24 h, and the growth of the bacterium around the antibiotic discs was observed, then the quantitative zone diameter measurements (in mm). The antimicrobial susceptibility testing was conducted in accordance with the Clinical and Laboratory Standard Institute and the European Committee on Antimicrobial Susceptibility Testing guidelines [26, 27].

### Evaluation of biofilm-forming capability

A static biofilm assay was conducted in U-type 96- plates using crystal violet staining according to Seper et al. with some modifications [28]. The respective isolates were cultured on nutrient agar (Oxoid^®^) and incubated for 24 h. Subsequently, bacterial growth was observed in tryptic soy broth, and the OD_595_ was adjusted to 0.02 [29]. Following this, 130 µl of each isolate dilution was seeded into the plate wells and incubated for 24 h at 37 °C. After incubation, the wells were rinsed with H2O distilled five times. Then, 160 µl of 0.1% crystal violet was added to each well for 10 min, followed by rinsing with distilled water. Subsequently, 210 µl of 96% ethanol was added to each well, and the OD_595_ was measured using the Infinite^®^ F50 Robotic (Ostrich) Microplate Reader to quantify the amount of biofilm The average OD_595_ of the sterile medium (negative control) was subtracted from all test results. The optical density of the cut-off (ODc) was determined as the average OD of the negative control plus 3 times the standard deviation (SD) of the negative control (mean OD negative controls+ (3 × SD)) [30]. We calculated the biofilm amount according to the method described by Stepanovic et al. [31]. Based on the OD values, the biofilm amount was categorized as follows, OD < ODc indicates a non-biofilm producer, ODc < OD < 2ODc indicates a weak biofilm producer, 2ODc < OD < 4ODc indicates a moderate biofilm producer, 4ODc < OD indicates a strong biofilm producer.

### Antibiotic susceptibility test

The susceptibility of Staphylococcal isolates to antimicrobial agents was assessed following the guidelines outlined by CLSI 2020 [26] using the disc diffusion method [32]. The antibiotics tested included cefepime (FEP-30), cefotaxime (CTX-30), tetracycline (TE-30), ampicillin/sulbactam (SAM-20), amoxicillin/clavulanic acid (AMC-30), penicillin G (P-10), ciprofloxacin (CIP-5), erythromycin (E-15), chloramphenicol (C-30), and vancomycin (VA-30) Bioanalyse^®^ (Bioanalysis Tibbi Malzemer, Turkey). The 24-hour bacterial growth was adjusted to OD_600_ of 0.5 MacFaddin [33] in tryptic soy broth. Subsequently, 100 µl of the broth was applied to Muller-Hinton agar plate and spread evenly. The antibacterial discs were then carefully positioned on the inoculated agar plate using sterile forceps, followed by an incubation period at 37 °C for 24 h. We examined the plates, and the clear zones of inhibition were measured using a standard ruler. The results were interpreted according to the guidelines provided by the Clinical and Laboratory Standard Institute [26] to determine the sensitivity of each isolate to the tested antibiotics, classifying them as resistant, intermediate, or sensitive. Antibiotic resistances were classified as multidrug-resistant (MDR) is resistance to at least one antibiotic from each of three categories of selected antimicrobial compound families (MDR >3) and pan-drug resistant (PDR) is resistant to all agents in all antimicrobial categories [34].

The criteria for selection (Methicillin-Resistant *S. aureus*) were the most antibiotics resistance and the strongest biofilm former isolates. Accordingly five strains of MRSA were selected for further molecular identifications and antibacterial activities.

### Detection of virulence and antibiotic resistance genes by polymerase chain reaction (PCR)

Molecular characterization was carried out by polymerase chain reaction (PCR). MRSA virulence genes (*icaA*, *icaD*, *clfA*, and *LukED*) and antibiotic resistance genes (*mecA*, *mecC*, and *blaZ*) were detected. The encoding of virulence and antibiotic-resistant genes was performed using forward and reverse primer sets as described in Table 1 [35–40]. Extraction of DNA according to QIAamp DNA mini kit instructions (Catalogue no.51304).

**Table 1:** Target genes, primers, sequences, cycling conditions, and amplicon sizes of MRSA isolates

Amplifications (35 cycles)									
Gene	Primer sequence (5’-3’)	Length of amplified product	Primary denaturation	Secondary denaturation	Annealing	Extension	No. of cycles	Final extension	Reference
**Virulence genes**									
*icaA*	CCT AAC TAA CGA AAG GTA G	1315 bp	94˚C 5 min.	94˚C 30 s.	49˚C 1 min.	72˚C 1 min.	35	72˚C 12 min	[35]
	AAG ATA TAG CGA TAA GTG C								
*icaD*	AAA CGT AAG AGA GGT GG	381 bp	94˚C 5 min.	94˚C 30 s.	49˚C 40 s.	72˚C 40 s.	35	72˚C 7 min.	
	GGC AAT ATG ATC AAG ATA								
*lukED*	TAGGCAAATCATCAGTTGCTTCAT	516 bp	94˚C 5 min.	94˚C 30 s.	56˚C 40 s.	72˚C 45 s.	35	72˚C 10 min.	[36]
	GTAGTTCTGTAACTTTCTTGTTT								
*clfA*	GCAAAATCCAGCACAACAGGAAACGA	638 bp	94˚C 5 min.	94˚C 30 s.	55˚C 40 s.	72˚C 45 s.	35	72˚C 10 min.	[37]
	CTTGATCTCCAGCCATAATTGGTGG								
**Antibiotic resistance gene**									
*mecA*	GTAGAAATGACTGAACGTCCGATA A	310 bp	94˚C 5 min.	94˚C 30 s.	50˚C 30 s.	72˚C 30 s.	35	72˚C 7 min.	[38]
	CCAATTCCACATTGT TTCGGTCTA A								
*mecC*	GCTCCTAATGCTAATGCA	304 bp	94˚C 5 min.	94˚C 30 s.	50˚C 30 s.	72˚C 30 s.	35	72˚C 7 min.	[39]
	TAAGCAATAATGACTACC								
*blaZ*	TACAACTGTAATATCGGAGGG	833 bp	94˚C 5 min.	94˚C 30 s.	50˚C 40 s.	72˚C 50 s.	35	72˚C 10 min.	[40]
	CATTACACTCTTGGCGGTTTC								

PCR amplification was performed using oligonucleotide primers (Metabion, Germany) that were utilized in a 25 µL reaction containing 12.5 µL of EmeraldAmp GT PCR Master Mix 2x premix (Takara^®^, Japan) No. RR310A kit, 1 µL of each primer, 5.5 µL PCR grade H_2_O, and 5 µL of DNA template. The Applied Biosystems 2720 Thermal Cycler was used for PCR. After amplification, the PCR products were separated by 1.5% agarose gel electrophoresis, with 20 µL of each product loaded in each gel slot. To determine the DNA fragment sizes, the Gene Ruler 100 bp ladder (Fermentas, Thermo) and Gelpilot 100 bp plus ladders (QIAGEN^®^, USA) were used as DNA molecular weight markers. The gel was photographed using a gel documentation system (Alpha Innotech^®^), and the data were analyzed using computer software (Automatic Image Capture, USA).

### Platelets collection, preparation and processing

Platelets were obtained from two male donors, aged 28 and 49 years, using the Trima Accel^®^ automated blood collection system-platelet apheresis unit. Both donors were healthy, nonsmoking donors, showed no signs of infection, and without recent antibiotic use [41]. Platelet samples were centrifuged at 4000 × g for 10 min (80 − 1 electric centrifuge, China) at room temperature. This process resulted in the separation of two layers, the upper, known as platelet-poor plasma (PPP), while the residual layer were withdrawn as platelet-rich plasma (PRP). Both platelets’ forms were collected in sterile tubes. Moreover, the blood cells (Platelets, red blood cells, and white blood cells) were counted for donor’s blood, platelets samples after collection and PRP preparations using a fully automated hematology analyzer (Abacus 380 cell counter, Diatron^®^, Hungary) [41]. finally, the activated fractions of platelet-rich plasma (PRP) ((2730 ± 70) × 10^9^ cells/L) were prepared as follows [42]:

1. PRP was activated by Calcium chloride (0.025 mol/l) (Biomed^®^) in ratio 5:1 to form APRP.
2. PRP was activated by Thromboplastin-s (Biomed^®^) in ratio 4:1 to form PRP-T.

Three activated fractions of PRP were prepared 20 min prior to use. After the formation of a clot, the liquid part (supernatant) was separated for use in antibacterial susceptibility tests [43].

### Antibacterial activities of platelets derivatives against the selected five MRSA strains in solid and liquid media

In solid media: Antibacterial activity was determined by using agar well diffusion method as described by Okeke et al. [33, 44]. Brifely50 µl of PPP (original plasma), (2730 ± 70 × 10^9^ cell/L) of each PRP, APRP, PRP-T and APRP-T were added separately to each well. Then the plates were incubated at 37 °C for 24 h. After incubation the plates were examined, and the inhibition clear zones were measured with standard ruler.

In liquid media: Serial dilutions were made from PRP, APRP, PRP-T, and APRP-T by using PPP as diluent, starting from the original cells concentration of (2730 ± 70) × 10^9^ cells/L. The dilutions were prepared as 10%, 20%, 40%, 60%, 80%, and 100%, as well as PPP (by using saline as a diluent). Each dilution (20 µl) was placed in a 96-well microtiter plate. Subsequently, 100 µl of bacteria, which had been incubated overnight and adjusted to OD_595_ of 0.01 [33] in tryptic soy broth, was added to all the wells. The negative control used sterile tryptic soy broth. Following an incubation period of 24 h at 37 °C, 40 µl of *p*-iodonitrotetrazolium (INT) (0.2 mg ml^− 1^, Sigma-Aldrich) was added to all the wells in the microtiter plate and incubated at 37 °C for 30 min [45]. The lowest concentration of the platelets derivatives that prevented a color change of INT were calculated as (cells/ L), as previously described by Lall et al. [46]. Consequently, the lowest concentration of the platelet’s derivatives were tested for its bacteriocidal activities by transferring 50 µl from each well to sterile nutrient agar plates and spreading it evenly using sterile glass beads. The plates were then incubated for 24 h at 37 °C. The lowest concentrations of the platelets derivatives cells that caused a complete bactericidal effect were also determined as (cells /L).

### Antibiofilm activity of platelets derivatives against MRSA strains

Biofilm inhibition assays were performed against some skin pathogens (MRSA) known to form strong static biofilms [29]. 130 µl of overnight bacterial suspensions adjusted to OD_595_ of 0.02 were seeded into the wells of a 96-well microtiter plate (U shape). The plates were then incubated at 37 °C for 24 h to allow biofilm development. Sterile TSB was used as a negative control, while bacterial suspension of MRSA used as positive control. After incubation, 30 µl of (2730 ± 70 × 10^9^cell/L) concentration of PRP, APRP, PRPT, APRPT, and PPP (original plasma) were added. The plates were then re-incubated for an additional 24 h. After the second incubation, the wells were washed five times with distilled water. To stain the biofilm, 160 µl of 0.1% crystal violet was added and left for 10 min. The wells were then rinsed with distilled water and solubilized by adding 210 µl of 96% ethanol. The amount of biofilm was quantified by measuring OD_595_ using the Infinite^®^ F50 Robotic (Ostrich) Microplate Reader.

Moreover, antibiofilm activity were scanned by fluorescence microscope, 96 microtiter plates (U shape) were prepared as previously described. The plates were stained by adding 160 µl of 0.1% crystal violet for 10 min. After staining, the plates were rinsed with distilled water and allowed to dry. They were then examined under a fluorescence microscope (LEICA DM2500, Germany) using a 10x lens (LEICA DFC450, Germany) under Emission wavelength equal 500–530 nm and Excitation wavelength equal 480 nm. Then the pictures were analyzed using software ImagJ to measure the average particle size and the surface area of the particles.

### Survival curve of MRSA strains in the presence of APRP and APRP-T

Microbial population at the initial and completion of the experiment was determined by plate count method at intervals of 4 h. 400 µl of 2730 ± 70 × 10^9^ cell/L of APRP and APRP-T (the most effective fractions) were added in sterile tubes separately. 2 ml of MRSA growth adjusted to OD_595_ of 0.01 [29] of overnight bacterial suspension were added. Tubes were incubated at 37 °C. Viable counts were determined by serial dilution method at initial time (0 times) and every 4 h up to 24 h. 10 µl of each dilution were spread on nutrient agar plates then incubated overnight at 37 °C. Plates counting between 30 and 300 colonies were counted [41] using Stuart^®^ colony counter (Stuart scientific, UK). Colonies number (CFU ml^− 1^) was plotted against time for each isolate.

### Statistical analysis

Data were analyzed using the Mann–Whitney U test or a Kruskal–Wallis test followed by post hoc Dunn’s multiple comparisons. Differences were considered significant at P values of ≤ 0.05. For all statistical analyses, GraphPad Prism version 8 was used.

## Results

### Clinical characteristics of patients

This study included a total of 102 patients with burns, abscesses, wounds discharge, hematomas, or other clinical signs of skin infection. Of these patients, 59 (57.84%) were males and 43 (42.16%) were females. Most patients were under the age of thirty, followed by the age between 30 and 60 years old, while the lowest distribution of skin disease were recorded in the age above 60 years old. Most participants had abscesses (93.14%). The clinical distribution of the studied cases is described in (Table 2).

**Table 2:** Distribution of patients according to age and gender among SSTIs diseases

Male		Female		Patient character /Age (year)	
57.84%	No = 59	42.16%	No = 43		
76.27	45	58.14	25	Below (30)	
13.56	8	39.53	17	Between (30–60)	
10.17	6	2.33	1	Above (60)	
**Prevalence of SSTIs disease**					
96.61	57	88.36	38	Abscess (*n* = 95)	
-	-	6.98	3	Burn (*n* = 3)	
3.39	2	2.33	1	Wounds discharge (*n* = 3)	
-	-	2.33	1	Hematoma (*n* = 1)	

No = number of patients, % percent of patients, SSTIs = Skin and soft tissue infections

### The frequency of SSTI isolates in the clinical samples under examination

**Fig. 1:**
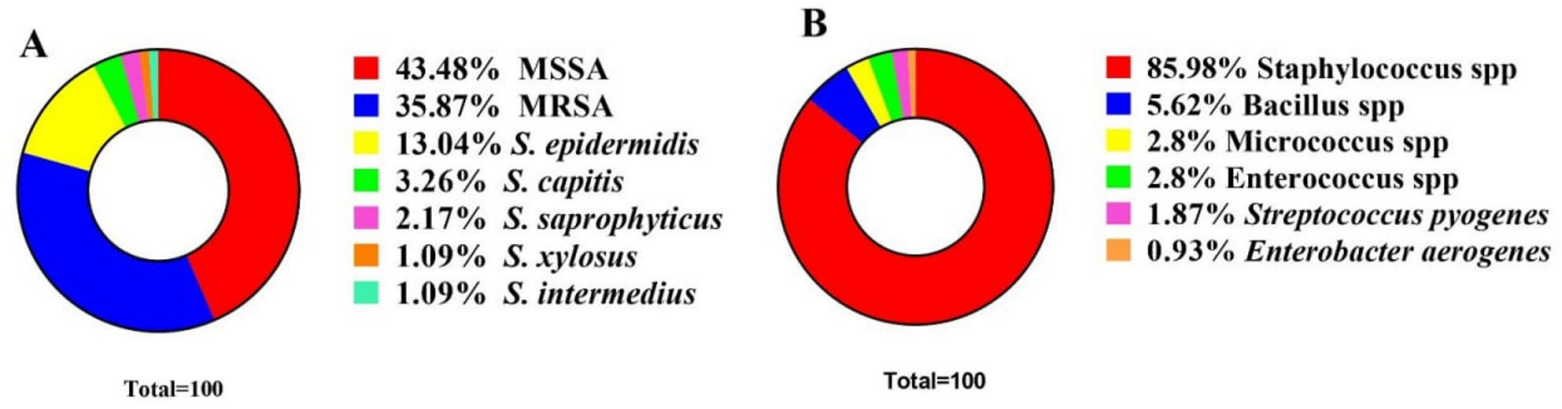
Frequency of *Staphylococcus aureus* among SSTIs isolates. (**A**) incidence of Staphylococcus spp isolates. (**B**) incidence of SSTIs isolates in pus samples

The most common predominant organisms among the 102 pus samples were *Staphylococcus* spp (85.98%), followed by *Bacillus* spp., *Micrococcus* spp and *Enterococcus* spp. *Streptococcus pyogenes* and *Enterococcus aerogenes* were the lowest percentages in this study (Fig. 1B). Among *Staphylococcus* spp *S. aureus* isolates were the most predominant with a percentage of 79.35% (43.48% MSSA; 35.87% MRSA), followed by *S. epidermidis.* While *S. capitis* and *S. saprophyticus* isolated in low percentages. Finally, *S. xylosus* and *S. intermedius* isolates were the lowest predominant by percentage as shown in Fig. 1A.

### Detection of some virulence factors of *Staphylococcus aureus*

100% of *S. aureus* strains tested positive for coagulase and DNase enzyme production. Fourteen of *S. aureus* strains were found to carry enterotoxin A (SEA) of them 12 MRSA strains, which is considered the most predominant toxin among *S. aureus* enterotoxins. Additionally, three strains of *S. aureus* two of them were MSSA were found to carry enterotoxin C (SEC), while enterotoxin D (SED) were the lowest appearance among *S. aureus* strains. Furthermore, four strains of *S. aureus* had the ability to produce more than one enterotoxin. Among these, two MRSA strains had a combination of SEA and SEC, and the other two MRSA strains had a combination of SEA and SED. Notably, SEB was absent in all strains (Table 3).

**Table 3:** *Staphylococcus aureus* enterotoxins serotyping

Staphylococcus aureus enterotoxins (SAE) serotyping						
Enterotoxin serotyping	*S. aureus* (*n* = 73)		MRSA (*n* = 31)		MSSA (*n* = 42)	
	No = 23	31.51%	No = 18	58.06%	No = 5	11.9%
Enterotoxin A (SEA)	14	19.18	12	38.7	2	4.76
Enterotoxin B (SEB)	0	0	0	0	0	0
Enterotoxin C (SEC)	3	4.11	1	3.23	2	4.76
Enterotoxin D (SED)	2	2.74	1	3.23	1	2.38
Enterotoxin A & C (SEA&SEC)	2	2.74	2	6.45	0	0
Enterotoxin A & D (SEA&SED)	2	2.74	2	6.45	0	0
Not-typed	50	68.49	13	41.94	37	88.1

No = number of isolates, % percent of isolates. Not-typed had no reaction with any of toxins included in the test

### Evaluation of biofilm-forming capability for *Staphylococcus* isolates

**Fig. 2:**
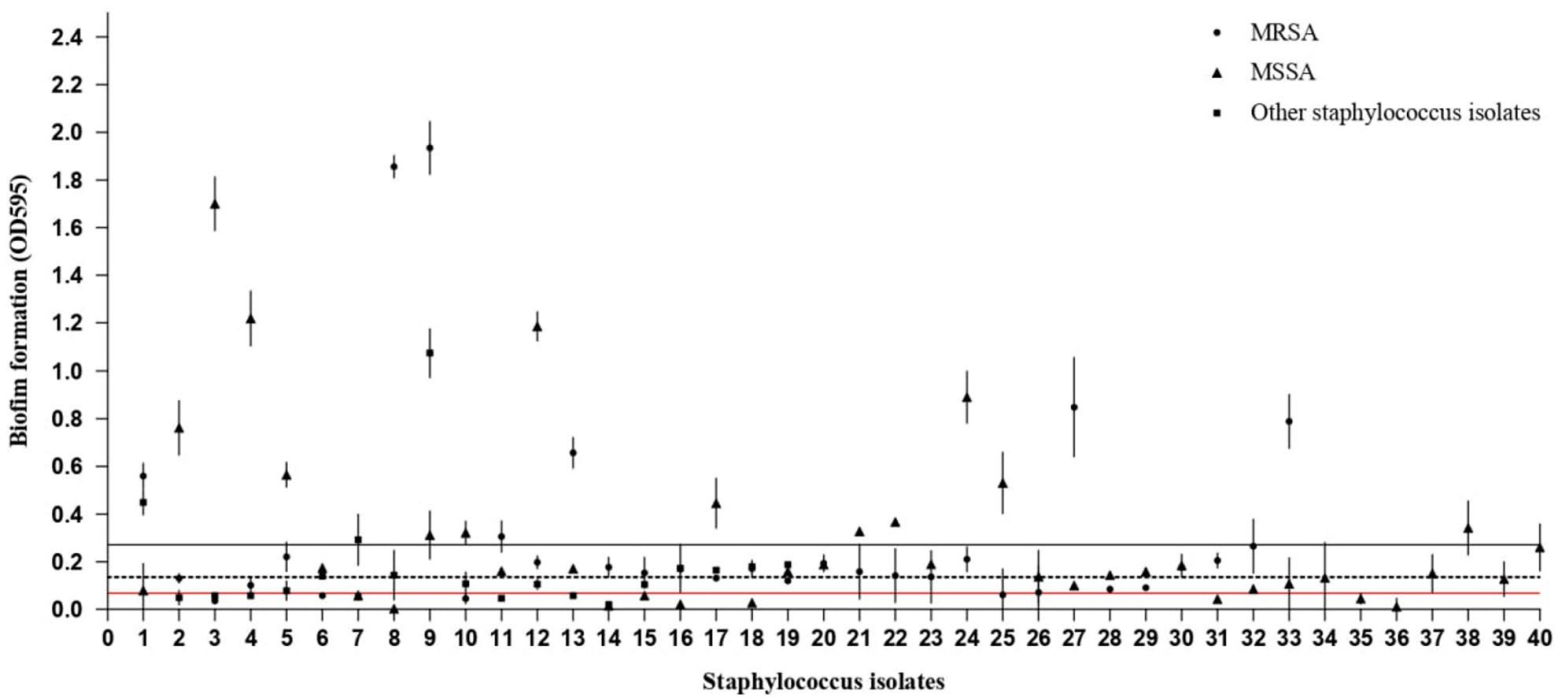
Biofilm formation values (OD 595) of staphylococcus isolates (*n* = 92) obtained by quantitative biofilm production assay. The OD cut-off used to distinguish weak and moderate biofilm producers from strong biofilm producers is 0.0677 (red line). Categories non-biofilm producers (OD ≤ 0.0677), weak biofilm producers 0.0677 < OD ≤ 0.1354 (dashed line), moderate biofilm producers 0.1354 < OD ≤ 0.2710 (black line), and strong biofilm producer 0.2710 < OD. (●) MRSA (1–23), (▲) MSSA (1–32), (■) Other staphylococcus isolates ((1–12) *S. epidermidis*, (13) *S. xylosus*, (14) *S. intermedius*, (15–16) *S. saprophyticus*, (17–19) *S. capitis*)

The majority of *Staphylococcus* isolates (34.78%) had the capability to form moderate biofilm formation, while 25.0% of isolates had the capability to form strong static biofilm. Additionally, 19.57% of isolates had the capability to form weak static biofilm. Among *S. aureus* strains, 27.24%, especially MSSA, exhibited strong biofilm ability. Furthermore, 21.21% MRSA strains demonstrated strong biofilm ability as shown in Table 4; Fig. 2.

**Table 4:** Distribution of biofilm degree among Staphylococcal isolates

Staphylococcus spp	% Biofilm producer (isolates)				
	Strong	Moderate	Weak	Non	Total (100%)
*S. aureus* *isolates*	27.4 (20)	35.62 (26)	19.18 (14)	17.8 (13)	73
MRSA	21.21 (7)	42.42 (14)	24.24 (8)	12.12 (4)	33
MSSA	32.5 (13)	30 (12)	15 (6)	22.5 (9)	40
*S. epidermidis*	25 (3)	16.67 (2)	25 (3)	33.33 (4)	12
*S. xylosus*	—	—	—	100 (1)	1
*S. intermedius*	—	—	—	100 (1)	1
*S. saprophyticus*	—	50 (1)	50 (1)	—	2
*S. capitis*	—	100 (3)	—	—	3
Total	25 (23)	34.78 (32)	19.57 (18)	20.65 (19)	92 (100)

% percent of isolates, MRSA methicillin resistant *S. aureus*, MSSA methicillin sensitive *S. aureus*

### Antibiotics susceptibility test

**Fig. 3:**
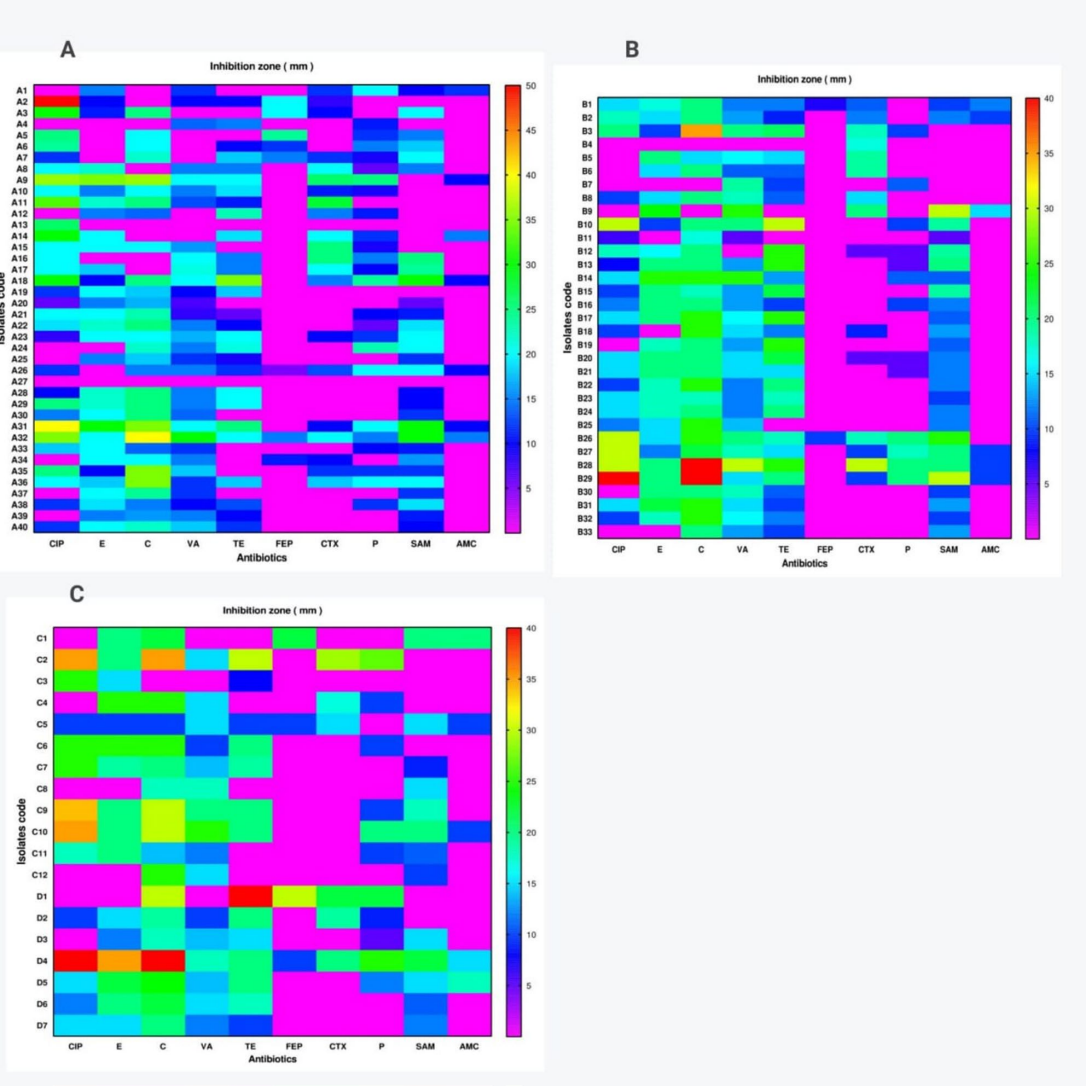
The heat-map displays each retrieved isolate’s antibiograms. (**A**) MSSA isolates, (**B**) MRSA isolates and (**C**) Other staphylococcus isolates. MSSA isolates (A1: A40), MRSA (B1: B33), *Staphylococcus epidermidis* (C1: C12), *S. xylosus* (D1), *S. intermedius* (D2), *S. saprophyticus* (D3, D4) and *S. capitis* (D5: D7) by antibiotic disc diffusion (mm). The intensity of colors indicates the numerical value of the inhibition zone (mm). ciprofloxacin (CIP-5), erythromycin (E-15), chloramphenicol (C-30), vancomycin (VA-30), tetracycline (TE-30), cefepime (FEP-30), cefotaxime (CTX-30), penicillin G (P-10), ampicillin/sulbactams (SAM-20), and amoxicillin/clavulanic acid (AMC-30). Antibiotic susceptibility breakpoint interpretations are based on CLSI 2020 standards

Ten standard antibiotics were used to test the antibiotic resistance of Staphylococcal isolates using a disk diffusion technique. The proportion of resistant isolates to examined antibiotics with distinct potency were presented as inhibition zone values (mm) in a heat-map as shown in Fig. 3. All MSSA strains demonstrated the highest resistance against amoxicillin/clavulanic acid and penicillin G. Additionally, 87.5% (*n* = 35) and 70% (*n* = 28) of MSSA strains were resistant to cefepime and cefotaxime, respectively. All MRSA strains were resistant to cefepime, amoxicillin/clavulanic acid, and penicillin G, while 75.8% exhibited resistance to ciprofloxacin and cefotaxime. 48.5% of MRSA strains were vancomycin resistant.

All strains of *Staphylococcus epidermidis* (*n* = 12) showed maximum resistance to penicillin G. Additionally, 91.67% of *S. epidermidis* strains were resistant to cefepime and amoxicillin/clavulanic acid. Similarly, all strains of *S. xylosus*,* S. saprophyticus*,* S. capitis*, and *S. intermedius* demonstrated maximum resistance to penicillin G and amoxicillin/clavulanic acid.

Nearly all isolates (98–100%) were MDR, with rare PDR detected in MSSA (2.5%). The other Staphylococcal isolates (100%) were Multidrug resistance (MDR ≥ 3) (Table 5).

**Table 5:** MDR pattern of Staphylococcal isolates from patients attending to Qena general hospital

Isolates (*n* = 92)	Resistance pattern number (%)										
	R0	R1	R2	R3	R4	R5	R6	R7	*R* ≥ 8	MDR (≥ 3)	PDR
***Staphylococcus aureus*** **(*n*** **= 73)**	0	0	1(1.4)	3(4.1)	6(8.2)	12(16.4)	21(28.8)	19(26)	11(15.1)	72(98.6)	1(1.4)
MRSA (33)	0	0	0	2(6.1)	1(3.0)	7(21.2)	10(30.3)	8(24.2)	5(15.2)	33(100)	0
MSSA (40)	0	0	1(2.5)	1(2.5)	5(12.5)	5(12.5)	11(27.5)	11(27.5)	6(15)	39 (97.5)	1(2.5)
**Other** ***Staphylococcus spp.*** **(*n*** **= 19)**	0	0	0	1	2	1(5.3)	7(36.8)	6(31.6)	2(10.5)	19(100)	0
*S. epidermidis* (12)	0	0	0	0	2(16.7)	1(8.3)	3(25)	4(33.3)	2(16.7)	12(100)	0
*S. xylosus* (1)	0	0	0	0	0	0	1(100)	0	0	1(100)	0
*S. saprophyticus* (2)	0	0	0	1(50)	0	0	0	1(50)	0	2(100)	0
*S. capitis* (3)	0	0	0	0	0	0	2(66.7)	1(33.3)	0	3(100)	0
*S. intermedius* (1)	0	0	0	0	0	0	1(100)	0	0	1(100)	0

R0; no resistance, R1; resistance to one antibiotic, R2; resistance to two antibiotics, R3; resistance to three antibiotics, R4; resistance to four antibiotics, R5; resistance to five antibiotics, R6; resistance to six antibiotics, R7; resistance to 7 antibiotics, *R* ≥ 8; resistance to more than or equal eight antibiotics.; MDR (≥ 3): multidrug resistance (for more than or equal to 3 antibiotics). PDR: pandrug-resistant (resistant to all antibiotics). MSSA: methicillin-sensitive *Staphylococcus aureus;* MRSA: methicillin-resistant *Staphylococcus aureus*

Most of (17.4–100%) the resistant strains were strong biofilm producers, 3.1–100% were moderate biofilm producers, while 0–69.6% of susceptible strains were identified as strong biofilm producers and 0–87.5% were moderate biofilm producers as shown in Table 6.

**Table 6:** Correlation between antibiotic resistant/susceptible strains and biofilm formation ability by phenotypic method

Biofilm producer (Isolates)																										
No	Antibiotics	Number tested	Strong (23)						Moderate (32)						Weak (18)						Non-Biofilm producer (19)					
			Sensitive		Intermediate		Resistant		Sensitive		Intermediate		Resistant		Sensitive		Intermediate		Resistant		Sensitive		Intermediate		Resistant	
			n	%	n	%	n	%	n	%	n	%	n	%	n	%	n	%	n	%	n	%	n	%	n	%
1	CIP-5	92	7	30.4	4	17.4	12	52.2	6	18.8	1	3.1	25	78.1	6	33.3	1	5.6	11	61.1	8	42.1	5	26.3	6	31.6
2	E-15	92	2	8.7	13	56.5	8	34.8	3	9.4	24	75.0	5	15.6	0	0	13	72.2	5	27.8	2	10.5	8	42.1	9	47.4
3	C-30	92	16	69.6	3	13.0	4	17.4	28	87.5	3	9.4	1	3.1	12	66.7	2	11.1	4	22,2	13	68.4	1	5.3	5	26.3
4	VA-30	92	10	43.5	0	0	13	56.5	16	50	0	0	16	50	10	55.6	0	0	8	44.4	11	57.9	0	0	8	42.1
5	TE-30	92	8	34.8	2	8.7	13	56.5	11	34.4	5	15.6	16	50	6	33.3	2	11.1	10	55.6	8	42.1	4	21.1	7	36.8
6	FEP-30	92	4	17.4	0	0	19	82.6	0	0	0	0	32	100	0	0	1	5.6	17	94.4	1	5.3	1	5.3	17	89.4
7	CTX-30	92	0	0	2	8.7	21	91.3	1	3.1	2	6.3	29	90.6	1	5.6	3	16.7	14	77.8	3	15.8	10	52.6	6	31.6
8	P-10	92	0	0	0	0	23	100	0	0	0	0	32	100	0	0	0	0	18	100	0	0	0	0	19	100
9	SAM-20	92	11	47.8	2	8.7	10	43.5	8	25	10	31.3	14	43.7	7	38.9	3	16.7	8	44.4	7	36.8	1	5.3	11	57.9
10	AMC-30	92	1	4.3	0	0	22	95.7	0	0	0	0	32	100	0	0	0	0	18	100	0	0	0	0	19	100

Number of antibiotics (No), number of isolates (n), percent of isolates (%), ciprofloxacin (CIP-5), erythromycin (E-15), chloramphenicol (C-30), vancomycin (VA-30), tetracycline (TE-30), cefepime (FEP-30), cefotaxime (CTX-30), penicillin G (P-10), ampicillin/sulbactam (SAM-20), and amoxicillin/clavulanic acid (AMC-30). Antibiotic susceptibility breakpoint interpretations are based on CLSI 2020 standards

### Detection of virulence and antibiotic-resistance genes by polymerase chain reaction (PCR)

**Fig. 4:**
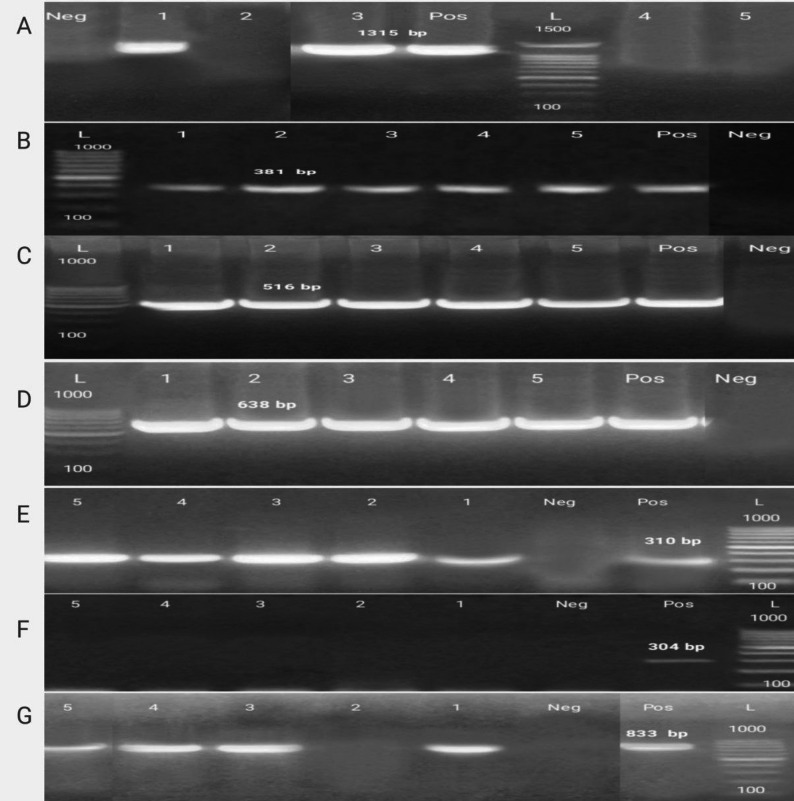
1.5% Agarose gel electrophoresis of uniplex PCR of virulence and antibiotic-resistant genes for characterization of MRSA isolates. (**A**) *icaA* (1315 bp), (**B**) *icaD* (381 bp), (**C**) *LukED* (516 bp), and (**D**) *clfA* (638 bp) virulence genes. (**E**) *mecA* (310 bp), (**F**) *mecC* (304 bp) and (**G**) *blaZ* (833 bp) antibiotic-resistant genes Lane L: Gel Pilot 100 bp plus ladder (cat. no. 239045) supplied from QIAGEN (USA) as molecular size DNA marker. Lane Pos: positive control of MRSA gene confirmed by reference laboratory for quality control. Lane Neg: negative control. Lane 1–5: MRSA isolates

The current investigation includes five MRSA strains due to their ability to form strong biofilm and their high resistance to antibiotics. These strains were examined for four virulence genes (*icaA*,* icaD*,* LukED*, and *clfA*) and three antibiotic-resistant genes (*mecA*,* mecC*, and *blaZ*) using PCR. 100% of MRSA strains carried the *icaD*,* LukED*, and *clfA* virulence genes while *icaA* was detected in 40% of strains (Table 7; Fig. 4).

**Table 7:** Antibiotic-resistant genes, and virulence genes by PCR for MRSA strains

MRSA strains	Virulence genes				Antibiotic-resistant genes		
	icaA	icaD	LukED	clfA	mecA	mecC	blaZ
1	+	+	+	+	+	-	+
2	-	+	+	+	+	-	-
3	+	+	+	+	+	-	+
4	-	+	+	+	+	-	+
5	-	+	+	+	+	-	+

(+): Positive) reaction, (-): Negative reaction, (*ica A*,* D*):Intercellular adhesion genes, *(LukED)*: Leucocidin ED gene, (*clfA*): Clumping Factor A gene, (*mecA*,* C*): Meticillin resistance gene, (*blaZ*) Beta-lactams resistant gene

Additionally, *mecA* and *blaZ* antibiotic-resistant genes were detected in 100% and 80% of MRSA strains, respectively. while *mecC* resistant gene was not detected in any of MRSA strain. The most frequently detected genes in all strains were *mecA*, *icaD*,* LukED*, and *clfA*.

### Antibacterial activity of platelet rich plasma (PRP)

#### Determination of platelets count

Platelet, red blood cell, and white blood cell counts were assessed in the donor’s whole blood, platelet samples after collection, and platelet-rich plasma (PRP) using a fully automated hematology analyzer (Abacus 380 cell counter, Diatron, Hungary). The complete blood counts of the donors were examined. As shown in Table 8, the complete blood count (CBC) revealed normal values. PRP contained ~ 5-fold higher platelet concentration, with reduced RBCs and WBCs In PRP, platelets were concentrated at a level of 2730 ± 70 × 10^9^ cells/L, which is considered 100%. The concentrations of RBCs and WBCs were 0.11 ± 0.1 × 10^12^ cells/L and 0.92 ± 1.22 × 10^9^ cells/L, respectively.

**Table 8:** Donors blood analysis for platelets, RBCs and WBCs

CBC	Donor (1)			Donor (2)		
	Before collection	After collection	PRP	Before collection	After collection	PRP
RBCs (10^12^/L)	5.62	0.02	0.04	5.47	0.05	0.18
WBCs (10^9^/L)	7.65	0.05	0.05	8.09	0.16	1.78
Platelets (10^9^/L)	256	1444	2680	285	1397	2779
PCT (%)	0.23	1.12	2.10	0.23	1.04	2.58
MPV (fl.)	9.1	7.7	7.8	8.0	7.5	9.3
PDWc (%)	37.9	36.6	36.9	37.9	36.4	41.8
P-LCC (10^9^/l)	73.0	286	556	78.0	330	1105
P-LCR (%)	28.65	19.97	20.74	27.19	23.61	39.75

CBC; Complete blood count. RBCs: Red blood corpuscles. WBCs: White blood cells. PCT: Plateletcrit (Volume occupied by platelets in the blood as a percentage). MPV: Mean platelet volume. PDWc: Platelet Distribution Width. P-LCC: Platelet large cell count. P-LCR: Platelet large cell ratio. All results of donors were in reference range

### Antibacterial effect of PPP, PRP and activated fractions of PRP in solid media

The antibacterial activity of PPP, PRP, APRP, PRP-T and APRP-T were evaluated against selected MDR MRSA strains using the well diffusion method (Table 9). The results showed that APRP and APRP-T were the most effective PRP fractions in suppressing bacterial growth exhibiting high potency. APRP and APRP-T demonstrated significant levels of antibacterial activity against MRSA strains, with inhibition zones ranging from 11.7 to 18 mm and 11.0 to 16.33 mm respectively. On the other side, all of the examined bacteria were resistant to PPP, PRP, and PRP-T.

**Table 9:** Antibacterial effect of PPP, PRP and PRP activated fractions on MRSA pathogens

MRSA	Inhibition zone (mm) (In solid media)		Antibacterial activities (cells /L) × 10^9^ (In liquid media)			
			Inhibition		killing	
	APRP	APRP-T	APRP	APRP-T	APRP	APRP-T
1	18 ± 1	16.33 ± 1.53	2730 ± 70	2730 ± 70	-	-
2	-	11.33 ± 0.58	2730 ± 70	2180 ± 30	-	2730 ± 70
3	11.7 ± 1.53	12.7 ± 2.08	2730 ± 70	2730 ± 70	-	-
4	12 ± 2	11 ± 1	2730 ± 70	2730 ± 70	-	-
5	16.7 ± 1.53	-	2730 ± 70	2730 ± 70	-	-

PPP: platelet poor plasma, PRP: platelet rich plasma, APRP: activated platelet rich plasma with CaCl_2_ (in ratio 5:1), PRP-T: PRP activated with thromboplastin (in ratio 4:1), APRP-T: APRP activated with thromboplastin (in ratio 4:1), (–): no effect

### Antibacterial effect of PPP, PRP and activated fractions of PRP in liquid media

APRP and APRP-T exhibited a bacteriostatic effect against some MDR SSTIs MRSA strains (Table 9) at cells concentrations of 2730 ± 70 × 10^9^ cell/L and 2180 ± 30 × 10^9^ cell/L. However, except for APRP-T, none of these substances had a bactericidal effect. APRP-T showed a bactericidal effect against MRSA (2) at cells concentration of 2730 ± 70 × 10^9^ cell/L.

### Antibiofilm activity of PPP, PRP and activate fractions of PRP by microtiter plate dye staining assay

**Fig. 5:**
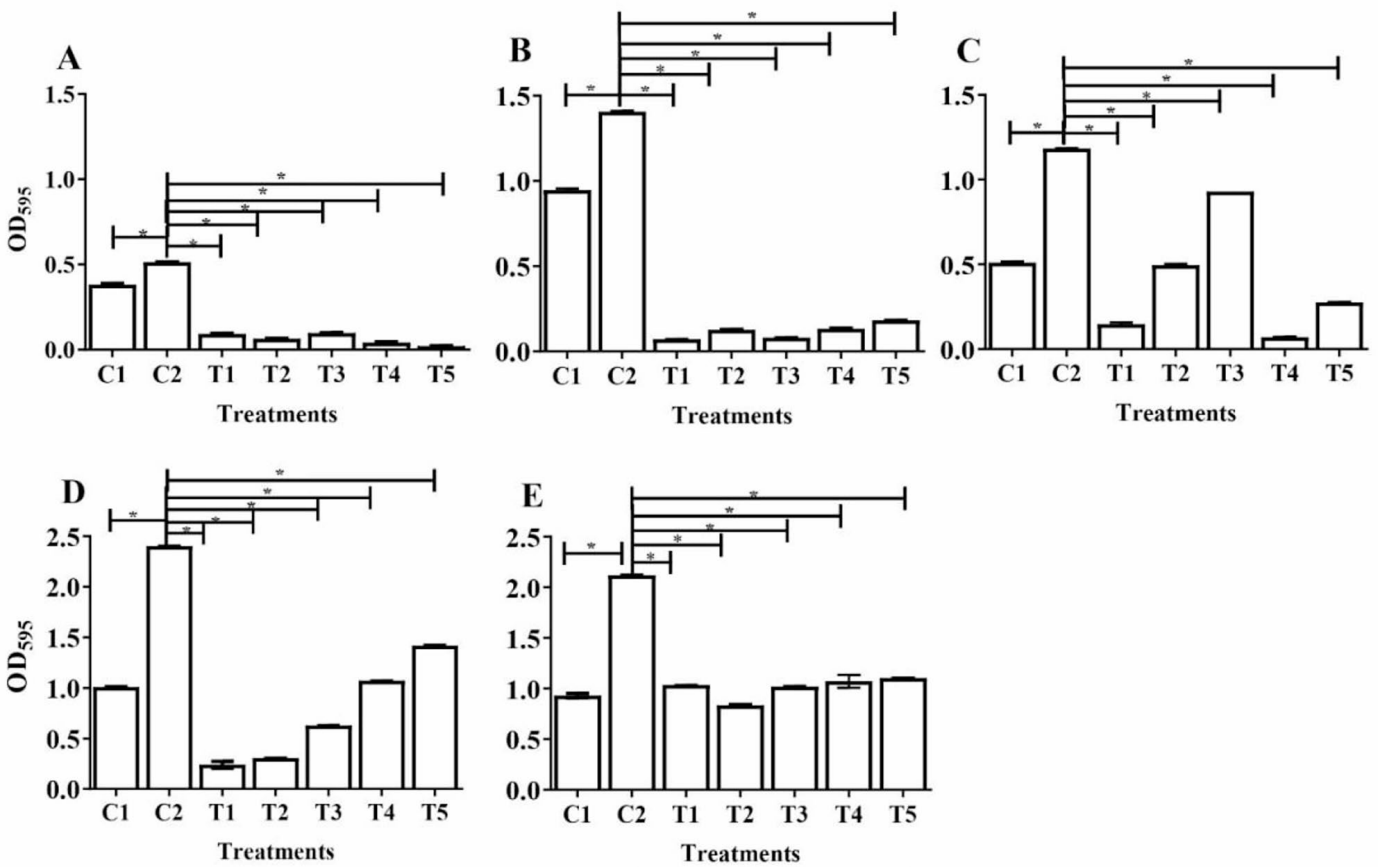
Impact of PPP (T1), PRP (T2), APRP (T3), PRP-T (T4) and APRP-T (T5) on biofilms of selected MRSA isolates. MRSA 1 (**E**), MRSA 2 (**B**) MRSA 3 (**C**), MRSA 4 (**D**), and MRSA 5 (**A**). PPP, PRP, APRP, PRP-T, or APRP-T were added after MRSA biofilms had grown for 24 h. The addition of MRSA broth served as a control after 24 h (C1), 48 h (C2) and biofilm production were measured by crystal violet staining after an extra 24 h, and the OD595 was then calculated. The medians from a minimum of eight separate measurements are displayed. The interquartile range is shown by the error bars. Asterisks denote meaningful variations between the data sets (*P* < 0.05; Kruskal–Wallis’s test and post hoc Dunn’s multiple comparisons)

All five platelet derivatives showed strong antibiofilm activity against MRSA strains. APRP-T (T5) and PRP-T (T4) were the most effective overall, reducing biofilm formation by 95.7% and 91.5%, respectively, particularly against MRSA (5). MRSA (2) was the most susceptible strain, with biofilm reductions ranging from 87.1 to 95%, especially when treated with PPP (T1). PRP-T (T4) was most effective against MRSA (3) (94% reduction), while PPP (T1) and PRP (T2) showed high activity against MRSA (4), reducing biomass by 90% and 87.3%. MRSA (1) was moderately affected, with reductions between 42.2 and 60.5%. Overall, PPP (T1) performed best against MRSA (2) and (4), PRP (T2) and APRP were highly effective against MRSA (2), PRP-T (T3) against MRSA (2, 3, 5), and APRP-T (T5) showed the strongest inhibition against MRSA (5) (Fig. 5).

### Antibiofilm detection by fluorescence microscope

**Fig. 6:**
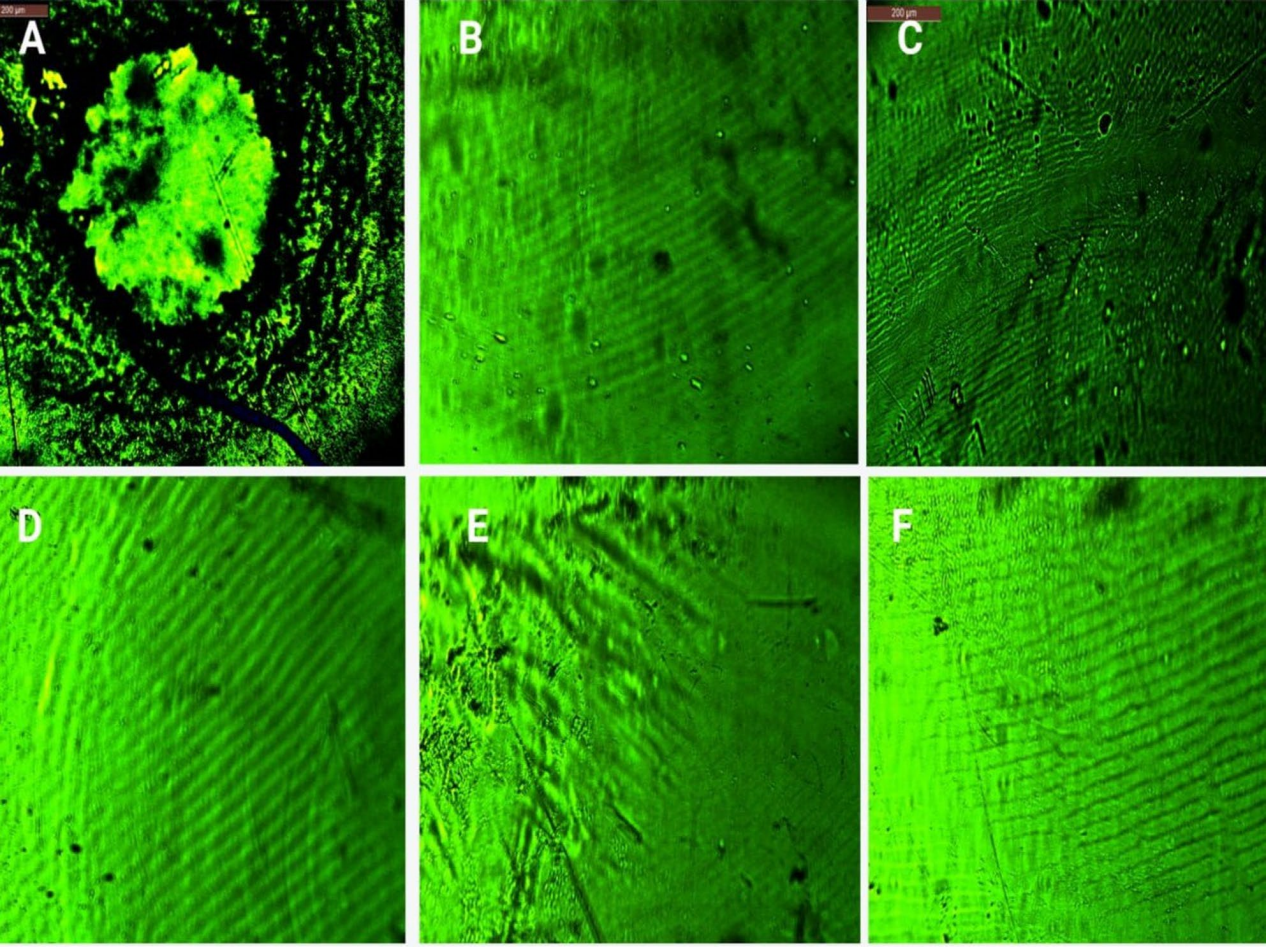
Fluorescence microscope pictures under lens 10x of biofilm formation of selected MRSA (2) in the presence of PPP, PRP and PRP active fractions. (**A**) control, (**B**) PPP, (**C**) PRP, (**D**) APRP, (**E**) PRP-T, (**F**) APRP-T

**Fig. 7:**
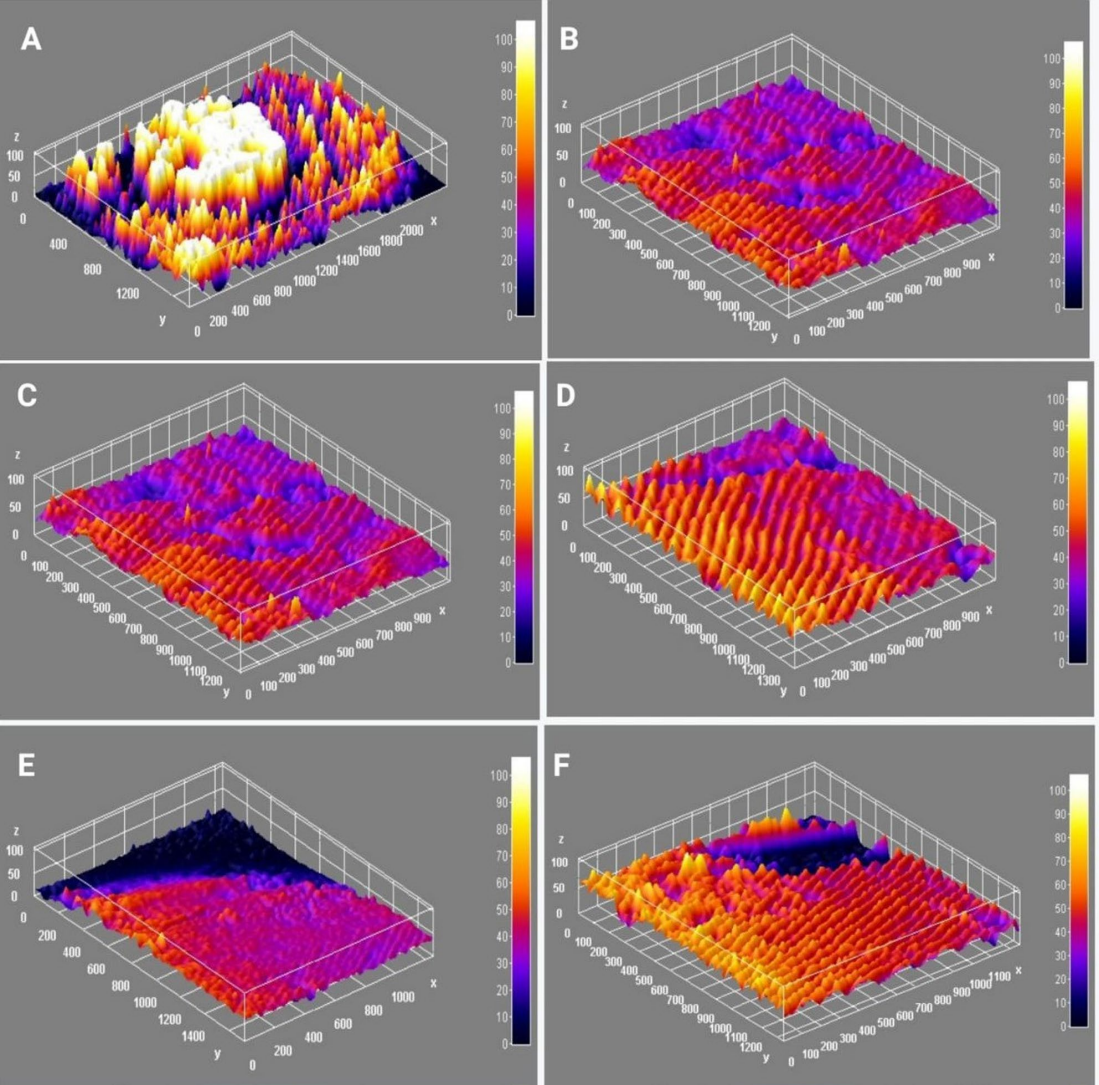
The interactive 3D surface plot for FM pictures by Imagj software of biofilm formation of MRSA (2) in the presence of PPP, PRP and PRP active fractions with fire legend. (**A**) control, (**B**) PPP, (**C**) PRP, (**D**) APRP, (**E**) PRP-T, (**F**) APRP-T

A fluorescence microscope was used to observe the general morphology of biofilms formed by MRSA. This technique, known as FM, was used to assess the antibiofilm effect of PPP, PRP, and PRP derivatives compared to a control of bacterial biofilm without antimicrobials. Figure 6 shows the images obtained by FM of MRSA (2) pictures. The FM pictures revealed a notable decrease in bacterial cell clumps in the presence of PPP, PRP, and PRP active fractions, as compared to the control. The analysis of the FM pictures was performed using ImageJ software, which utilized particle analysis threshold to measure the particle count, average particle size, and surface area of particles (Table 10). There was a significant decrease in particle count and size in the presence of PPP, PRP, and PRP active fractions as described in Fig. 7. The interactive 3D surface plot for FM pictures with fire legend showed the interactive 3D surface plot for FM pictures, it demonstrated a notable decrease in peak size in the presence of PPP, PRP, and PRP active fractions compared to the bacterial control.

**Table 10:** Particle analysis of biofilm florescence photos by ImagJ software

Fractions	Count	Total area	Average size	Area %	Mean	Mode	lntDen
Control	19,766	2,978,978	150.712	60.607	7.653	6.004	8585.645
PPP	209	4513	21.593	0.092	152.550	151.368	3979.560
PRP	180	4058	22.544	0.083	121.806	122.161	3853.639
APRP	51	4152	81.412	0.084	152.942	152.941	14865.235
PRP-T	273	6702	24.549	0.136	158.139	157.546	4360.879
APRP-T	122	616	5.049	0.013	255	255	1287.541

Particles analysis of biofilm fluorescence microscope pictures by ImageJ software by using particles analysis threshold of biofilm formation of MRAS (2) in the presence of PPP, PRP and PRP active fractions. Total area: Area of selection in square pixels; Area %: The percentage of pixels in the image or selection that have been highlighted in red (particles) Mean: Average gray value (the sum of the gray values of all the pixels divided by the number of pixels); Mode: Modal gray value (Most frequently occurring gray value); lntDen: Integrated density (the product of Area and Mean Gray Value)

### Survival curve of MRSA strains in the presence of APRP and APRP-T

**Fig. 8:**
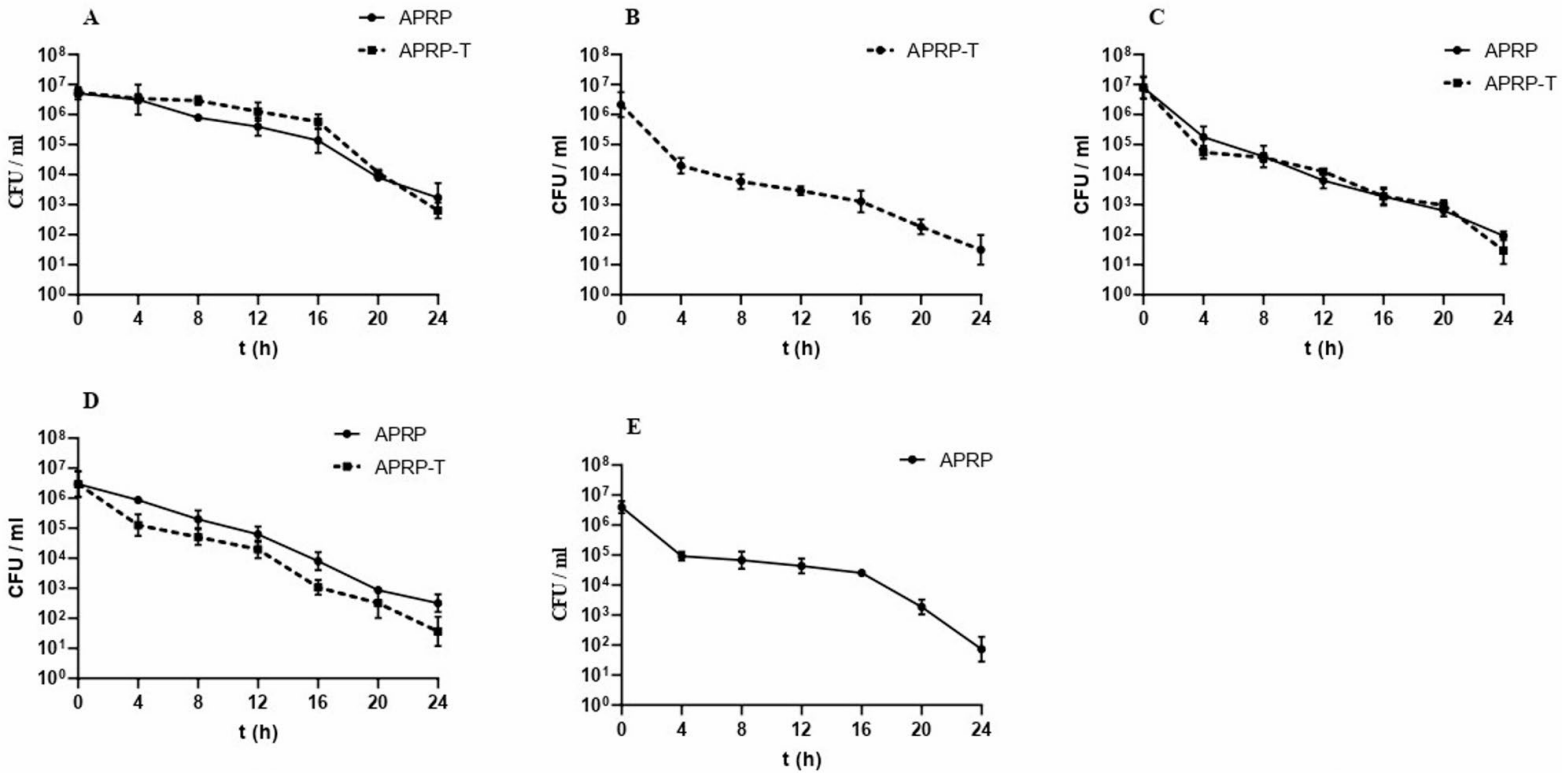
Survival curve of MRSA isolates in presence of APRP and APRP-T. Shown are the median CFU of MRSA (1–5) (**A-E**) in the presence of APRP (solid line) or APRP-T (dashed line) over time. The medians from a minimum of three separate measurements are displayed. Each data set’s interquartile range for each time point is shown by the error bars

Figure 8 illustrates the time–kill kinetics of APRP and APRP-T against logarithmic-phase cultures of MRSA isolates (A–E). Both preparations exhibited a time-dependent reduction in viable cell counts (CFU/mL) across all tested strains. A consistent decline in bacterial viability was observed within the first 4 h of treatment, with a pronounced decrease evident after 24 h. Quantitative analysis of the mean CFU values ± standard deviation (SD) indicated statistically significant reductions (*p* < 0.05) after 24 h compared to the initial inoculum in all strains. APRP-T demonstrated a slightly greater inhibitory effect than APRP in most strains (A, C, and D), as reflected by lower mean CFU counts and smaller error bars at 24 h, indicating both stronger and more consistent bacteriostatic activity. In strains where only one preparation was tested (B and E), a final CFU levels approaching or below 10³ CFU/mL, confirming a substantial suppression of bacterial growth.

Overall, these findings confirm that both APRP and APRP-T exert significant bacteriostatic effects against MRSA isolates, with APRP-T displaying marginally superior performance in reducing bacterial viability over 24 h.

## Discussion

A cutaneous abscess is a deep, pus-filled infection that forms when bacteria enter the skin through hair follicles, cuts, or wounds [47]. Due to increasing antibiotic resistance, higher antibiotic doses are often required to treat these infections [48]. Abscesses are the most common type of skin and soft tissue infection (SSTI) [49]. In a study at Qena General Hospital in Egypt, abscesses accounted for 93.14% of SSTI cases, with the highest prevalence in individuals under 30 years old (68.63%) and a higher occurrence in males (57.84%), findings consistent with earlier studies [49, 50]. Most infections (95.3%) were caused by a single pathogen, similar to previous reports [50]. *Staphylococcus aureus* was identified as the predominant pathogen, responsible for about 80% of SSTIs, mainly presenting as abscesses and cellulitis [51]. In the recent study, *S. aureus* accounted for 79.35% of isolates, including 43.48% methicillin-sensitive (MSSA) and 35.87% methicillin-resistant (MRSA) strains. Other identified bacteria included *S. epidermidis*, *S. capitis*, *S. saprophyticus*, *S. xylosus*, S. intermedius, *Streptococcus pyogenes*, *Enterococcus spp*. (*E. faecalis* and *E. faecium*), *Micrococcus* spp. (*M. roseus* and *M. luteus*), *Bacillus* spp. (*B. licheniformis*, *B. subtilis*, and *B. pumilus*), and *Enterobacter aerogenes*, consistent with earlier SSTI studies [50–55].

*S. aureus* has the ability to synthesize enzymes such as coagulase and deoxyribonuclease [56]. Coagulase, in particular, plays a crucial role in abscess formation by altering host defense mechanisms and promoting coagulation, thereby contributing to *S. aureus* pathogenesis [57]. All *S. aureus* isolates in the study were found to produce coagulase and DNase, which further increased their virulence. *S. aureus* utilizes a wide range of virulence factors, including toxins, to establish an infection in the host [58]. Among these factors, heat-stable enterotoxins are particularly notable. Staphylococcal enterotoxins (SEs) not only cause toxic shock-like syndromes and food poisoning but also act as superantigens that stimulate T-cell proliferation and interfere with the activity of immune cells, especially those present in or recruited to the skin [59]. In a recent study five prototypic SEs (ABCDE) were examined. SEA was the most common (19.2%), especially among MRSA strains. A few strains of *S. aureus* were positive for Sect. (4.1%) and SED (2.7%). In addition to some strains (5.48%) could produce more than one toxin. SEAD more frequency detected in MRSA, while SEAC is most frequent in MSSA, this in agreement with the results of Sila et al. [60]. Staphylococcal enterotoxins (SEs) cause toxic shock-like syndromes and have been implicated in food poisoning. But SEs also act as super antigens that stimulate T-cell proliferation. Also interfere in the activity of immune cells, particularly those which reside in or are recruited to the skin [59].

Recognition of the fact that bacterial biofilm may play a role in the pathogenesis of disease has led to an increased focus on identifying bacteria that may be biofilm-related. Biofilm infections are typically chronic in nature, as biofilm-residing bacteria can be resilient to the immune system, antibiotics, and other treatments [61]. The ability to form static biofilm was performed by crystal violet staining method. About 79.4% of *Staphylococcus* isolates had the ability to form biofilm. 82.2% of *S. aureus* were biofilm formers (strong 27.4%, moderate 35.6% and weak 19.2%). The majority of MRSA (42.4%) formed moderate biofilm, while 21.2 and 24.2% of MRSA isolated formed strong and weak biofilm. Our results agreed with the results of Piechota et al. [62]. The ability to develop biofilms varies among different *S. aureus* strains. Multiple environmental factors, including nutrients, antibacterial agents, pH, shearing force, temperature, and so on can induce stress responses and can profoundly affect the life cycle stages of biofilm formation, including initial attachment, maturation, and detachment [63]. Biofilms are more resistant to antibiotics than planktonic cells due to the multi-level protection conferred by the extracellular matrix (which hinders the penetration of antibiotics), altered metabolic states and growth rate [64]. Furthermore, the biofilm formation ability of MRSA strains, together with their often associated multidrug-resistance profile, enhances the overall resistance, resulting in chemotherapeutic failure [65]. In addition, the proximity of bacterial cells within the biofilm promotes horizontal genetic transfer, conjugation and mobilization of antimicrobial resistance genes [66].

The antibiotic susceptibility of Staphylococcal isolates was determined using a disk diffusion method. A total of 98.9% of the strains were found to be multidrug-resistant (MDR). The emergence of penicillin-resistant *S. aureus* occurred in the late 1940s, and by the mid-1950s, penicillin resistance had become so prevalent that the antibiotic was no longer effective for the treatment of infections [67]. Resistance to β-lactam antibiotics is a significant challenge when treating *S. aureus* infections. However, this pathogen can also develop resistance to other antibiotics, including vancomycin, which is crucial for severe MRSA infections [67]. All MRSA strains displayed the highest resistance to cefepime, amoxicillin/clavulanic acid, and penicillin G. Additionally, 75.8% of the MRSA strains were resistant to ciprofloxacin and cefotaxime. Furthermore, 48.5% of the MRSA strains showed resistance to vancomycin. These findings are consistent with the results reported by Kobayashi et al. [68], who found that MRSA strains were resistant to penicillin, ciprofloxacin, chloramphenicol, tetracycline, and vancomycin by 97.4%, 86.1%, 59%, 61.5%, and 20.1%, respectively. Mohanty et al. [50] also reported that 50% of MRSA strains were vancomycin resistant. Sienkiewicz et al. [55] reported that *Staphylococcus* strains isolated from wound infections were highly resistant to various antibiotics, including β-lactams (penicillin), macrolides (erythromycin), lincosamides (clindamycin), tetracycline, fluoroquinolones (ciprofloxacin), aminoglycosides (gentamicin), and sulfonamides (trimethoprim-sulfamethoxazole). *S. aureus* clinical strains exhibited higher resistance to recommended antibiotics compared to *S. epidermidis* and *S. xylosus* strains, but all of them were resistant to penicillin.

In this study, we investigated the presence of virulence and antibiotic-resistant genes in some MRSA strains. Among the MRSA strains, all of them carried the *icaD*,* LukED*, and *clfA* virulence genes. Additionally, the *icaA* gene was detected in 40% of the strains. In terms of antibiotic resistance, the *mecA* gene was detected in all MRSA strains, while the *blaZ* gene was detected in 80% of the strains. The *mecC* resistant gene was not detected in any MRSA strain. Our findings are higher than those reported by Piechota et al. [62], who found that only 15.4% of *S. aureus* strains carried the *icaAD* genes, and small percentage (3.1%) of strains harbored only the *icaA* gene. Mohanty et al. [50] reported a higher prevalence of the *mecA* gene in *S. aureus*, with 63.3% of strains testing positive.

The *icaA* and *icaD* genes play a crucial role in intercellular adhesion and the formation of bacterial multilayers in biofilm production. These genes are associated with both slime and biofilm formation in *S. aureus* [9]. Biofilm formation is closely linked to the expression of *ica* genes [69]. Our results align with a study by Szczuka et al. [70], which found that *ica-*positive MRSA biofilms were thicker and had a more compact architecture compared to *ica-*negative isolates. Furthermore, *clfA* is a major staphylococcal fibrinogen-binding protein [71]. This protein, along with the extracellular fibrinogen binding protein, blocks phagocytosis by depositing fibrinogen on the bacterial surface [72].

The antibacterial activity of platelets preparations was investigated against selected MDR MRSA isolates using the well diffusion method (solid media), and in liquid media as well. The five plasma and PRP fractions evaluated were carefully selected to identify the main antimicrobial component(s). All plasma and PRP preparations were prepared from the same donors to limit the impact of individual donor variations [41]. PPP was tested to define the antimicrobial role of plasma innate or humoral immunity system [41]. Our results showed that PPP had no antibacterial effect. Also, non-activated PRP was used as control of the starting material and to determine if platelets affect bacterial growth [41]. Our results showed that PRP (non- activated) had no antibacterial effect. Activation of PRP is necessary to form a fibrin matrix for platelet attachment and adhesion. Activation of PRP is also crucial for the bio activation of platelets-rich gel (PRG) that results in degranulation, the release of the substances and growth factors that contribute to the wound healing cascade, and the antibacterial effects of PRP [14]. Beyond direct antimicrobial action, APRP supports tissue repair and immune-cell recruitment, which helps restore barrier integrity and reduce bacterial colonization [73]. Our results confirmed this finding, where APRP (PRP activated by Calcium Chloride) and APRP-T (PRP activated by Calcium Chloride and thromboplastin) were the most effective activated PRP fractions in suppressing bacterial growth with high potency by inhibition zone ranging from 11.0 to 18.0 mm and platelets counting causing bacterial inhibition range 2180 ± 30 and 2730 ± 70 × 10^9^ cell/L, while PRP-T (PRP activated by thromboplastin only) had no antibacterial effect. Bielecki et al. [74] showed that the susceptibility zones on Mueller–Hinton agar, platelet-rich gel showed antimicrobial activity against *S. aureus* and *E. coli* but not against *E. faecalis*. Inhibition zones produced by platelet-rich gel were between 6 and 24 mm (mean 9.83 mm) in diameter. Edelblute et al. [75] study showed that human platelet gel supernatants were moderately significant decrease in *S. aureus*. L-PRP exhibited leukocyte subtype mediated in vitro antibacterial activity against MRSA, MSSA, *E. faecalis*, and *P. aeruginosa*, but no antibacterial effect was demonstrated for *E. coli*, and *K. pneumonia* [42]. The study of Li et al. [76] showed that both EPG (PRP without activation) and PRG (PRP activated by Calcium Gluconate/thrombin) significantly reduced bacterial count of *S. aureus* compared to PPP. Moojen et al. [77] revealed that antimicrobial effect of PRP is thanks to platelets rather than leukocytes by showing PRP without thrombin did not have antimicrobial effect. PRP needs to be activated by thrombin to develop antibacterial effect. Thrombin activates only platelets, but it has no effect on leukocytes in PRP. Growth factors such as PDGF, TGF-β, EGF, VEGF, IGF-1, FGF, HGF, and various anti-inflammatory peptides released by the activated platelets are responsible for the wound healing [78]. Despite the heterogeneity in the studies in terms of PRP preparation, treatment targets, and experimental methods, most studies consistently show that PRP is most effective against Gram-positive bacteria, including the difficulty treating Gram-positive species MRSA [75, 77, 79, 80]. PRP is both bactericidal and bacteriostatic. Depending on the bacterial load, host status, bacterial type, and the overall “dose” of PRP, it may achieve the MIC and overcome the rate of bacterial growth enough to stop replication. If the dose of PRP is insufficient, it may slow growth but be subsequently overcome as the antimicrobial aspects of PRP are depleted over time. Several authors suggested that a continued dose of PRP over the wound healing time is more effective than a single application [80].

PRP had the ability to inhibit MRSA growth and biofilm attachment [48]. Antibiofilm activity of PPP, PRP and PRP active fractions against SSTIs MRSA isolates biofilm were performed by two methods: dye staining assay and fluorescence microscope assay. All five fractions had significant antibiofilm potency. All MRSA strains biofilm OD especially MRSA (2) were reduced in the presence of PPP, PRP and PRP active fractions. Różalski et al. [81] The authors reported that “expired” platelets and their lysates significantly reduced the population of *S. aureus* and decreased metabolic activity of biofilm formation. Burnouf et al. [41] reported that PPP and PRP have similar total protein, fibrinogen, immunoglobulins, and albumins, while PG has depleted fibrinogen and coagulation factors. The antibiofilm activity of PPP and PRP derivatives is related to the content of innate plasma in decreasing the metabolic activity of biofilm formation. It is possible that a higher cell count of platelets does not always ensure a high concentration of growth factors in the PRP-based final product [14].

SSTIs MRSA pathogen strains were examined to determine the effects of PRP (platelet-rich plasma) on bacterial growth. The results showed a continuous decrease in the number of pathogen cultures cfu after 4 h. There was a sharp detectable growth decrease after 24 h, indicating that APRP and APRP-T had bacteriostatic actions against SSTIs MRSA isolates. The PRP dispersion demonstrated a significant decrease in MRSA growth for 24 h, confirming its bacteriostatic properties [48]. Previous studies have also shown the antibacterial effects of platelet preparations. Burnouf et al. [41] found that platelet preparations exhibited antibacterial effects as early as 3 h. Li et al. [82] showed that PRP, when activated, reduced the colony formation units (CFU) of MRSA within the first 2 h compared to the control, whereas PPP (platelet-poor plasma) did not show any effect on these bacterial strains. Intravia et al. [83] demonstrated that both PRP-LP (low platelet concentration) and PRP-HP (high platelet concentration) significantly decreased bacterial growth of *S. aureus*,* S. epidermidis*, and MRSA at 8 h. The study concluded that the antibacterial activity of PRP-LP and PRP-HP was not substantially affected by the differences in platelet and WBC (white blood cell) concentration, suggesting that the numbers of platelets and leukocytes do not directly impact antibacterial activity. Mariani et al. [43] found that *S. aureus* was significantly inhibited for at least 4 h, regardless of the preparation tested or the number of seeded bacteria. Three new molecules (NAP-2, SDF-1α, and IL-6) showed strong correlations with the growth inhibition of almost all the bacteria analyzed. Çetinkaya et al. [84] demonstrated that both PRP and PPP significantly suppressed the growth of MRSA as early as 1 h. The antimicrobial effect of PRP was more effective than PPP, but this difference was statistically significant only in MRSA during the first hours of the study. However, their results did not show a statistically significant correlation between the antibacterial effect of L-PRP (leukocyte-rich PRP) and platelet count [42].

These findings suggest that APRP and APRP-T could serve as promising adjunct therapies for MRSA-related skin and soft tissue infections (SSTIs), offering a biologically based approach to enhance infection control, promote tissue regeneration, and reduce antibiotic dependence. Our findings align with prior studies demonstrating PRP’s efficacy against Gram-positive bacteria [77, 79], though variability in preparation methods may account for discordant results. These differing results may be due to differences in study design, such as the preparation of PRP, variations in platelet counts, whether the platelets are activated or not, the yield of bacteria, and the antibiotic resistance profile of the bacteria. However, both in vitro and in vivo studies consistently show that there are no contraindications for the use of PRP on infected wounds [84]. Li et al. [76] and other authors describe the multiple roles of native platelets in host defense against infection. These roles include generating antimicrobial oxygen metabolites, facilitating complement fixation on bacteria, internalizing and clearing pathogens from the bloodstream, executing antibody-dependent cell cytotoxicity, potentiating antimicrobial mechanisms of leukocytes, and degranulating and releasing various cationic antimicrobial peptides such as VEGF, PDGF-BB, IGF-1, and TGF-β1. Platelets are known to contain several AMPs, such as thrombocidins, defensins, and platelet microbicidal proteins (PMPs). These peptides, derived from platelet α-granules, exhibit broad-spectrum activity against bacteria, fungi, and some viruses. Platelet microbicidal proteins (PMPs) are small cationic peptides that can disrupt microbial membranes, enhancing innate immune defense at wound sites [76, 82, 85–88]. Platelet preparations have been shown to increase the concentration of different growth factors, including PDGF, TGF-β1, VEGF, IGF-1, IL-6, IL-8, EGF, and IL-1β, which promote the wound healing process [76, 81, 89]. Platelet-Derived Growth Factor (PDGF): Stimulates fibroblast proliferation and chemotaxis, Transforming Growth Factor-β (TGF-β) regulates collagen synthesis and modulates inflammation, Vascular Endothelial Growth Factor (VEGF) promotes angiogenesis and tissue perfusion. Insulin-Like Growth Factor-1 (IGF-1) supports cell survival and tissue remodeling [92–94]. Upon activation, platelets release alpha-granule-derived antimicrobial peptides such as Platelet Factor 4 (PF4/CXCL4), RANTES (CCL5), thymosin β-4 and others, which disrupt bacterial membranes and exert direct bactericidal activity [15]. A key study found that PRP activated with CaCl₂ released significantly higher levels of EGF and PDGF-AB and induced a progressive release of GFs up to 24 h [90, 91]. The potential clinical implications extend to wound care, dermatology, and surgical infection management—areas where MRSA remains a significant therapeutic challenge. By integrating PRP-based strategies into current treatment protocols, clinicians may improve healing outcomes and mitigate the growing issue of antibiotic resistance in chronic and postoperative wounds. Further clinical trials are warranted to validate these findings and optimize PRP formulations for routine clinical application.

## Conclusion

The antibiotic resistance of invading bacteria is a serious threat throughout the world, and large doses of antibiotics to eradicate drug resistant bacteria are needed. PRP, a biocompatible product, can be considered as an effective antibacterial preparation with activity against SSTIs especially abscess colonized bacteria (MRSA). In this study, PRP was investigated for its antimicrobial and antibiofilm ability against abscess colonized by MRSA. PRP were applied under different activation modes. The most effective active fractions were APRP (PRP activated with Calcium Chloride) and APRP-T (PRP activated with a combination of Calcium Chloride and thromboplastin). All fractions had antibiofilm ability (PPP, PRP and PRP active fractions). However, further in vitro and in vivo studies using clinical isolates are needed to assess whether the antimicrobial effects of PRP could have positive effects against other bacterial pathogens. We recommend careful examination of developing methods for storage and its effect on the PRP antibacterial activity and more studies in extracting the active components and concentrating it from PRP active fractions.

## Supplementary Information

Below is the link to the electronic supplementary material.

Supplementary Material 1

Supplementary Material 2

Supplementary Material 3

Supplementary Material 4

Supplementary Material 5

Supplementary Material 6

Supplementary Material 7

## Data Availability

Data is provided within the manuscript and supplementary information files.

## Abbreviations

PRP: Platelet-rich plasma
PPP: Platelet-poor plasma
APRP: Activated platelet rich plasma with CaCl2 (in ratio 5:1)
PRP-T: PRP activated with thromboplastin (in ratio 4:1)
APRP-T: APRP activated with thromboplastin (in ratio 4:1)
MDR: Multidrug-resistant bacteria
PDR: Pandrug-resistant (resistant to all antibiotics)
CoNS: Coagulase negative Staphylococci
CFU: Colony forming unit
SSTIs: Skin and soft tissue infections
INT: p-iodonitrotetrazolium
MIC: Minimum inhibitory concentration
MBC: Minimum bactericidal concentration
SEA: *S. aureus* enterotoxin A
SEB: *S. aureus* enterotoxin B
SEC: *S. aureus* enterotoxin C
SED: *S. aureus* enterotoxin D
CBC: Complete blood count
RBCs: Red blood corpuscles
WBCs: White blood cells
FM: Fluorescence microscope
MSSA: Methicillin-sensitive *Staphylococcus aureus*
MRSA: Methicillin-resistant *Staphylococcus aureus*
ica: Intercellular adhesion
LukED: Leucocidin ED
clfA: Clumping Factor A
