# Supplementary material for: Antibacterial and antibiofilm activity of platelet-rich plasma under different activation conditions against multidrug-resistant MRSA isolated from human skin abscesses
Source: BMC Biotechnol. 2025 Dec 8;25:137. doi: 10.1186/s12896-025-01078-x (PMC12690961; doi:10.1186/s12896-025-01078-x)
Supplement: Supplementary file 1 — Supplementary Material 1 [file 12896_2025_1078_MOESM1_ESM.docx]

**Supplement Table (1): Biochemical reactions of Staphylococcus spp. isolates**

| **Biochemical tests** | ***S. aureus*** | ***S. epidermidis*** | ***S. capitis*** | ***S. saprophyticus*** | ***S. intermedius*** | ***S. xylosus*** |
| --- | --- | --- | --- | --- | --- | --- |
| Catalase | + | + | + | + | + | + |
| Oxidase | - | - | - | - | - | - |
| Hemolysis | *β* | γ | γ | γ | γ | γ |
| Growth at 6.5 % NaCl | + | + | - | + | + | + |
| Growth at 10 ^o^C | - | - | - | - | - | - |
| Growth at 45 ^o^C | - | - | - | - | - | - |
| Esculin hydrolysis | - | - | + | + | + | + |
| Arginine decarboxylase | + | + | + | - | + | - |
| Hippurate hydrolysis | - | - | - | - | - | + |
| Sugar fermentation: | | | | | | |
| Lactose | - | - | - | - | - | - |
| Mannitol | + | - | - | - | - | - |
| Arabinose | - | - | + | - | + | + |
| Ribose | V | V | - | + | + | - |
| Sorbitol | - | - | - | - | - | - |
| Raffinose | - | - | - | - | - | - |

**(-): Negative, (+): Positive, (V): Variable.**
