# Supplementary material for: Antibacterial and antibiofilm activity of platelet-rich plasma under different activation conditions against multidrug-resistant MRSA isolated from human skin abscesses
Source: BMC Biotechnol. 2025 Dec 8;25:137. doi: 10.1186/s12896-025-01078-x (PMC12690961; doi:10.1186/s12896-025-01078-x)
Supplement: Supplementary file 2 — Supplementary Material 2 [file 12896_2025_1078_MOESM2_ESM.docx]

**Supplement Table (2): Biochemical reactions of other Gram positive cocci isolates**

| **Biochemical tests** | ***Streptococcus pyogenes*** | ***Enterococcus faecalis*** | ***Enterococcus faecium*** | ***Micrococcus roseus*** | ***Micrococcus luteus*** |
| --- | --- | --- | --- | --- | --- |
| Catalase | - | - | - | + | + |
| Hemolysis | *β* | γ | γ | γ | γ |
| Growth at 6.5 % NaCl | - | + | + | - | + |
| Growth at 10 ^o^C | - | + | + | + | - |
| Growth at 45 ^o^C | - | V | V | - | - |
| Esculin hydrolysis | V | + | + | V | V |
| Arginine decarboxylase | + | + | + | - | V |
| Hippurate hydrolysis | - | + | V | - | - |
| Sugar fermentation: | | | | | |
| Lactose | + | + | + | - | V |
| Mannitol | - | + | + | - | - |
| Arabinose | - | - | V | - | + |
| Ribose | - | + | - | + | V |
| Sorbitol | - | + | - | V | - |
| Raffinose | - | - | - | - | V |

**(-): Negative, (+): Positive, (V): Variable.**
