# Supplementary material for: Antibacterial and antibiofilm activity of platelet-rich plasma under different activation conditions against multidrug-resistant MRSA isolated from human skin abscesses
Source: BMC Biotechnol. 2025 Dec 8;25:137. doi: 10.1186/s12896-025-01078-x (PMC12690961; doi:10.1186/s12896-025-01078-x)
Supplement: Supplementary file 3 — Supplementary Material 3 [file 12896_2025_1078_MOESM3_ESM.docx]

**Supplement Table (3): Biochemical reactions of Bacillus spp*.* isolates**

| **Biochemical tests** | ***Bacillus subtilis*** | ***Bacillus licheniformis*** | ***Bacillus pumilus*** |
| --- | --- | --- | --- |
| Motility | + | + | + |
| Hemolysis | γ | γ | γ |
| Catalase | + | + | + |
| Oxidase | - | - | + |
| Gelatin liquefaction | + | + | - |
| Nitrate reduction | + | + | - |
| Arginine decarboxylase | - | - | - |
| Lecithinase | - | - | - |
| Esculin hydrolysis | + | V | + |
| Starch hydrolysis | + | + | + |
| Ornithine decarboxylase | - | - | - |
| Lactose | + | + | - |
| Sucrose | + | + | V |
| Arabinose | + | + | + |
| Mannitol | + | + | + |
| Xylose | + | V | + |
| Maltose | + | + | + |
| Sorbitol | + | + | - |
| Rhamnose | - | - | - |

**(-): Negative, (+): Positive, (V): Variable.**
