# Supplementary material for: Antibacterial and antibiofilm activity of platelet-rich plasma under different activation conditions against multidrug-resistant MRSA isolated from human skin abscesses
Source: BMC Biotechnol. 2025 Dec 8;25:137. doi: 10.1186/s12896-025-01078-x (PMC12690961; doi:10.1186/s12896-025-01078-x)
Supplement: Supplementary file 4 — Supplementary Material 4 [file 12896_2025_1078_MOESM4_ESM.docx]

**Supplement Table (4): Biochemical reactions of Gram-negative isolates**

| **Biochemical tests** | ***Enterobacter aerogenes*** |
| --- | --- |
| Motility | + |
| Indole | - |
| Methyl red | *-* |
| Voges-Proskuaer | + |
| Citrate utilization | + |
| Urease | - |
| H_2_S production | - |
| Nitrate reduction | + |
| Gelatin liquefaction | - |
| Ornithine decarboxylase (ODC) | + |
| L-lysine decarboxylase (LDC) | + |
| Arginine decarboxylase (ADH) | - |
| β- galactosidase (ONPG) | - |
| Lactose | + |
| Sucrose | + |
| Dulcitol | - |
| Salicin | + |
| Arabinose | + |
| Inositol | + |
| Xylose | + |

**(-): Negative, (+): Positive**
