# Supplementary material for: Antibacterial and antibiofilm activity of platelet-rich plasma under different activation conditions against multidrug-resistant MRSA isolated from human skin abscesses
Source: BMC Biotechnol. 2025 Dec 8;25:137. doi: 10.1186/s12896-025-01078-x (PMC12690961; doi:10.1186/s12896-025-01078-x)
Supplement: Supplementary file 5 — Supplementary Material 5 [file 12896_2025_1078_MOESM5_ESM.docx]

**Supplement Table (5): Prevalence of SSTIs bacteria among different diseases.**

| Isolates | Abscess | burning | Wounds | Hematoma | Total |
| --- | --- | --- | --- | --- | --- |
| Number (%) | | | | | |
| *Staphylococcus aureus* | 70 (65.4) | 2 (1.87) | 1 (0.9) | - | 73 (68.2) |
| MRSA | 32 (29.9) | - | 1(0.9) | - | 33 (30.8) |
| MSSA | 38 (35.5) | 2(1.87) | - | - | 40 (37.4) |
| Other Staphylococcus spp. | 17 (15.9) | - | 1 (0.9) | 1 (0.9) | 19 (17.6) |
| *S. epidermidis* | 11 (10.3) | - | - | 1 (0.9) | 12 (11.2) |
| *S. xylosus* | - | - | 1 (0.9) | - | 1(0.9) |
| *S. intermedius* | 1 (0.9) | - | - | - | 1(0.9) |
| *S. saprophyticus* | 2 (1.87) | - | - | - | 2 (1.87) |
| *S. capitis* | 3 (2.8) | - | - | - | 3 (2.8) |
| *Streptococcus*  *pyogenes* | 1 (0.9) | - | 1 (0.9) | - | 2 (1.87) |
| Enterococcus spp. | 2 (1.87) | 1 (0.9) | - | - | 3 (2.8) |
| *E. faecalis* | 2 (1.87) | - | - | - | 2 (1.87) |
| *E. faecium* | - | 1 (0.9) | - | - | 1(0.9) |
| Micrococcus spp. | 3 (2.8) | - | - | - | 3 (2.8) |
| *M. roseus* | 1 (0.9) | - | - | - | 1(0.9) |
| *M. luteus* | 2 (1.87) | - | - | - | 2 (1.87) |
| Bacillus spp. | 6 (5.6) | - | - | - | 6 (5.6) |
| *B. licheniformis* | 4 (3.7) | - | - | - | 4 (3.74) |
| *B. subtilis* | 1 (0.9) | - | - | - | 1(0.9) |
| *B. pumilus* | 1 (0.9) | - | - | - | 1(0.9) |
| G-ve bacilli | 1 (0.9) | - | - | - | 1(0.9) |
| *E. aerogenes* | 1 (0.9) | - | - | - | 1(0.9) |
