# Supplementary material for: Antibacterial and antibiofilm activity of platelet-rich plasma under different activation conditions against multidrug-resistant MRSA isolated from human skin abscesses
Source: BMC Biotechnol. 2025 Dec 8;25:137. doi: 10.1186/s12896-025-01078-x (PMC12690961; doi:10.1186/s12896-025-01078-x)
Supplement: Supplementary file 6 — Supplementary Material 6 [file 12896_2025_1078_MOESM6_ESM.docx]

**Supplement Table (6) Antibiofilm activity**

| MRSA code | Bacterial control OD_595_ | | Treatments | | | | | | | | | | | | | | | |
| --- | --- | --- | --- | --- | --- | --- | --- | --- | --- | --- | --- | --- | --- | --- | --- | --- | --- | --- |
|  |  |  |  | PPP | |  |  | PRP | |  |  | APRP | |  | PRP-T | | APRP-T | |
|  | 24 h | 48 h | OD_595_ | | Reduction % | | OD_595_ | | Reduction % | | OD_595_ | | Reduction % | | OD_595_ | Reduction % | OD_595_ | Reduction % |
| 1 | 1.005 ± 0.013 | 2.118 ± 0.008 | 1.036±0.001 | | 51.1 | | 0.837±0.012 | | 60.5 | | 1.022±0.004 | | 51.8 | | 1.106±0.01 | 42.2 | 1.104±0.002 | 47.9 |
| 2 | 0.951 ± 0.002 | 1.406 ± 0.004 | 0.070±0.001 | | 95.0 | | 0.127±0.005 | | 91.0 | | 0.080±0.003 | | 94.3 | | 0.133±0.006 | 90.4 | 0.182±0.004 | 87.1 |
| 3 | 0.511 ± 0.002 | 1.185 ± 0.001 | 0.148±0.01 | | 87.5 | | 0.496±0.007 | | 58.1 | | 0.931±0.001 | | 21.4 | | 0.071±0.002 | 94.0 | 0.275±0.003 | 76.8 |
| 4 | 1.005 ± 0.003 | 2.404 ± 0.005 | 0.240±0.034 | | 90.0 | | 0.306±0.007 | | 87.3 | | 0.628±0.007 | | 73.9 | | 1.071±0.005 | 55.4 | 1.419±0.005 | 41.0 |
| 5 | 0.385 ± 0.004 | 0.515 ± 0.001 | 0.093±0.003 | | 81.9 | | 0.065±0.001 | | 87.4 | | 0.098±0.001 | | 81.0 | | 0.044±0.003 | 91.5 | 0.022±0.001 | 95.7 |
