# Supplementary material for: Antibacterial and antibiofilm activity of platelet-rich plasma under different activation conditions against multidrug-resistant MRSA isolated from human skin abscesses
Source: BMC Biotechnol. 2025 Dec 8;25:137. doi: 10.1186/s12896-025-01078-x (PMC12690961; doi:10.1186/s12896-025-01078-x)
Supplement: Supplementary file 7 — Supplementary Material 7 [file 12896_2025_1078_MOESM7_ESM.docx]

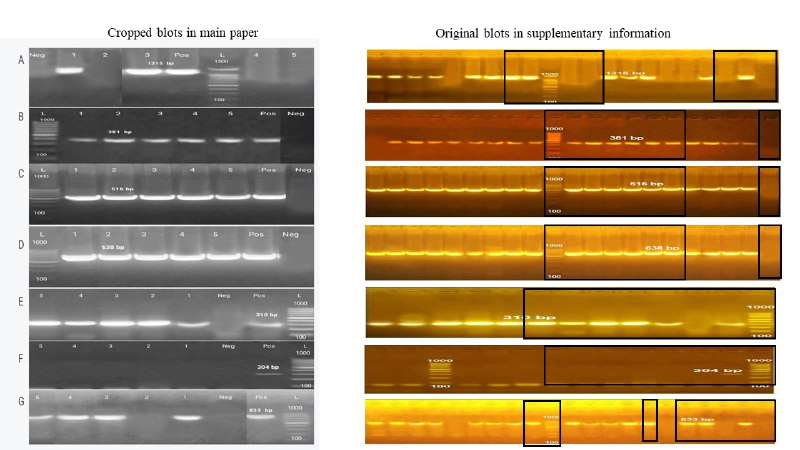


**Fig. 4** 1.5% Agarose gel electrophoresis of uniplex PCR of virulence and antibiotic-resistant genes for characterization of MRSA isolates.

Caption: (A) *icaA* (1315 bp), (B) *icaD* (381 bp), (C) *LukED* (516 bp), and (D) *clfA* (638 bp) virulence genes. (E) *mecA* (310 bp), (F) *mecC* (304 bp) and (G) *blaZ* (833 bp) antibiotic-resistant genes Lane L: Gel Pilot 100 bp plus ladder (cat. no. 239045) supplied from QIAGEN (USA) as molecular size DNA marker. Lane Pos: positive control of MRSA gene confirmed by reference laboratory for quality control. Lane Neg: negative control. Lane 1: MRSA (1). Lane 5: MRSA (2). Lane 6: MRSA (3). Lane 9: MRSA (4). Lane 10: MRSA (5).


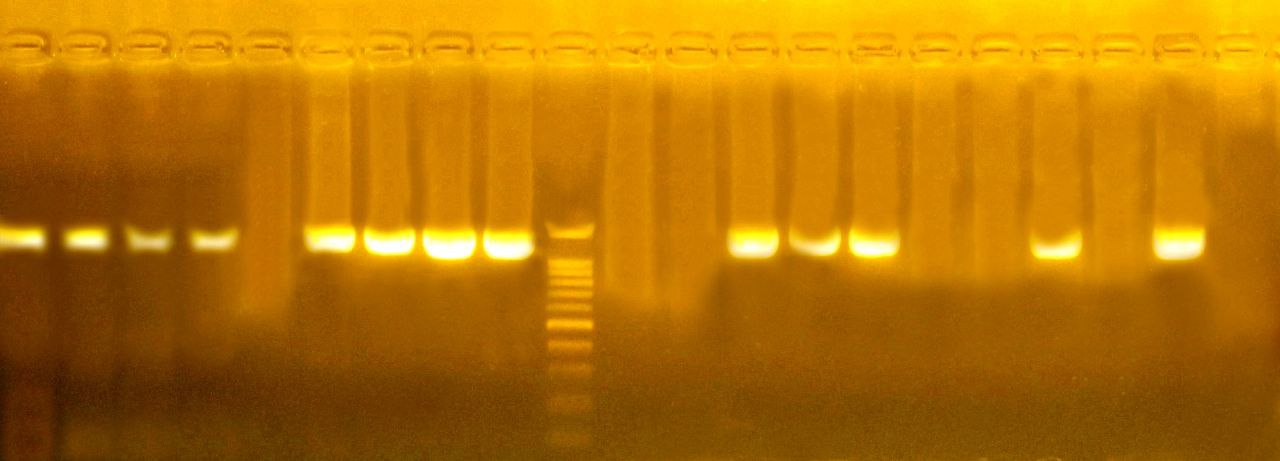


Original blots in supplementary information

Fig 4A

1315 bp

Fig 4B


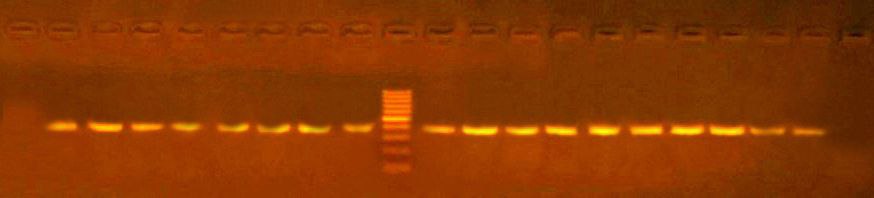


381 bp

Fig 4C


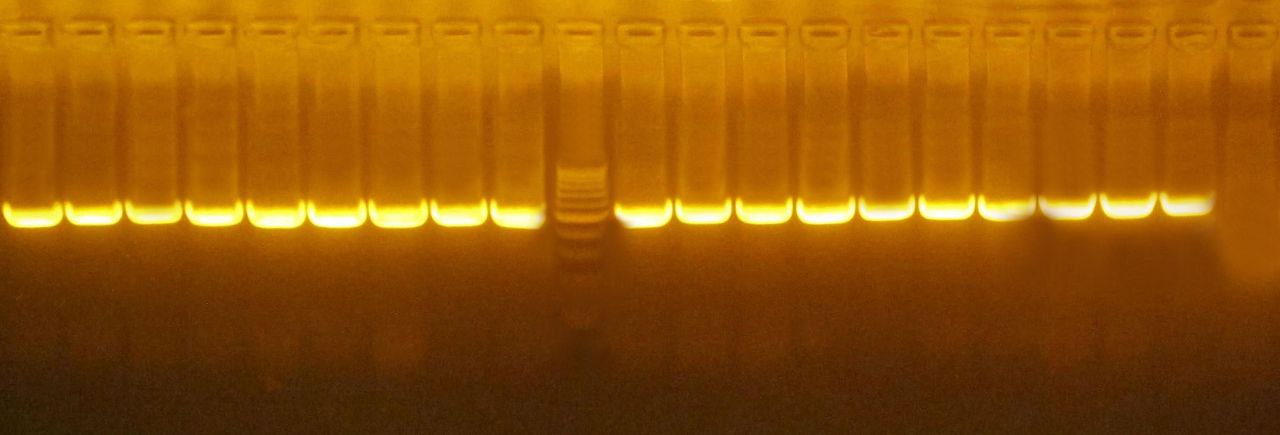


516 bp


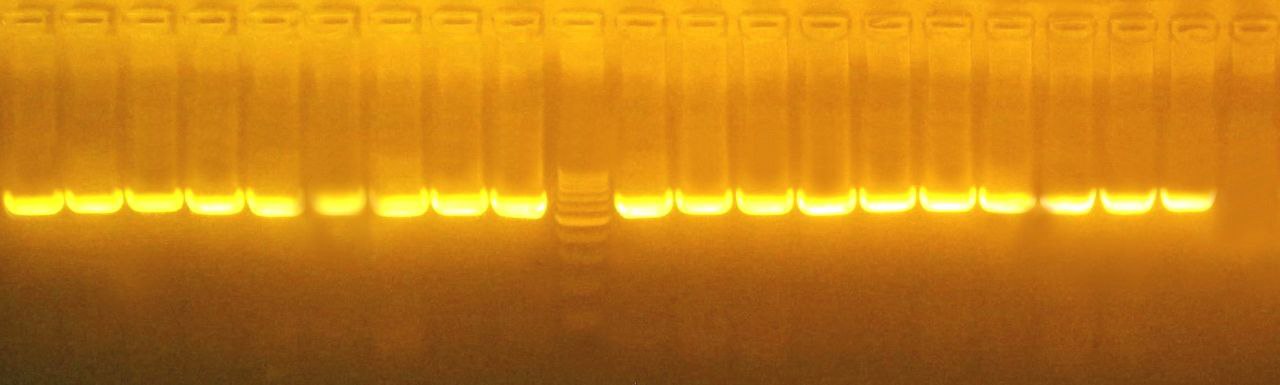


Fig 4D

638 bp

Fig 4E


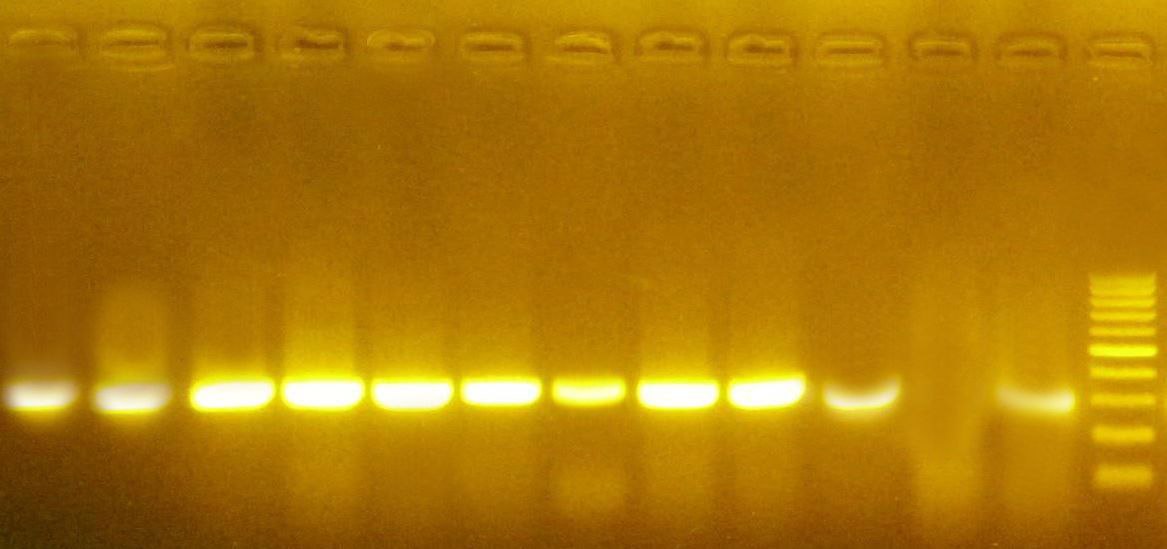


310 bp

Fig 4F


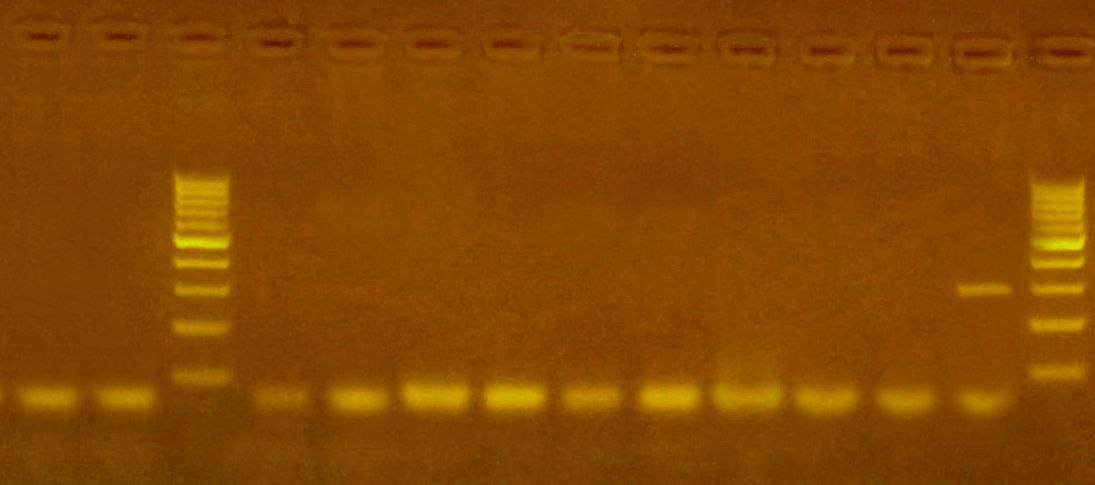


304 bp


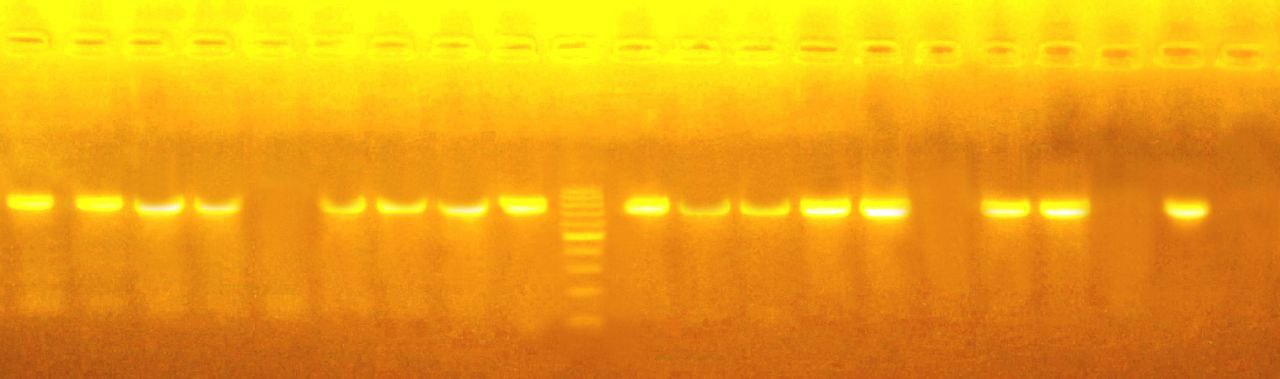


Fig 4G

833 bp
